# Quarterly Periscope, or Spirit of the Public Journals

**Published:** 1822-09-01

**Authors:** 


### Quarterly Periscope

OF

### PRACTICAL MEDICINE;

BEING THE

### Spirit of the Public Journals,

FOREIGN AND DOMESTIC.

*Paucis libris immorari et innutriri oportet, si velis aliquid trahere, quod in animo fideliter hæreat.* SENECA.

Duo vitia vitanda sunt in cognitionis et scientiæ studio. ***** Alterum est vitium, quod quidam nimis magnam operam conferunt in res obscuras atque difficiles, easdemque non necessarias. CICERO.

1. *Fever.** Dr. Armstrong's writings on fever have deservedly obtained such reputation for their author that, as is said of Lord Byron, "it is out of his power to write any thing that will sink." It is on this account that he must be doubly anxious not to write any thing that *deserves* to sink.

The present communication to our respected cotemporary, is an AVANT-COURIER to a forth-coming work, for which we shall look with much impatience. Dr. Armstrong states that, in 1819, he attended a patient labouring under an intermittent fever, which, in its progress, put on a remittent character, and ultimately a continued form and typhoid type, accompanied by very malignant symptoms. This case made a deep impression on his mind, and inclined him to ask, whether intermittent, remittent, and typhus fevers, might not possibly be modifications of one and the same disease. Up to this period Dr. A. had firmly believed that human contagion was the sole cause of genuine typhus fever; but a doubt having been excited, he determined, if possible, to divest his mind of all former bias, and investigate the subject ab origine. In the course of the three succeeding years, a very great number of typhus cases have fallen under his notice, and the result of his observations is a decided conviction "that what the Italians vaguely call *malaria*, and the English, as vaguely, *marsh effluvium*, is the primary source of typhus fever."

* Dr. Armstrong's Observations on the Origin, Nature, and Prevention of Typhus Fever. Med. Intelligencer, No. xxx. May 1822.

We have always considered, that the great epidemics which have ravaged this and other countries at different periods, derived their origin from *terrestrial exhalations*,* the operation of which was probably aided by certain states of the atmosphere, not yet ascertained; but that these fevers have propagated themselves afterwards by a *new source*—human contagion—has now been so fully proved, that nothing, we apprehend, can shake the foundation of the belief. Some of the ablest practitioners of the present day believe that genuine typhus fever, as described by Huxham and others, is now a rare disease; and a considerable portion of our readers will remember the celebrated discussion which took place at Guy's Hospital, between Drs. Curry, Clutterbuck, and Armstrong, on the subject of fever about four years ago, wherein Dr. Curry distinctly insisted on the terrestrial origin of what are termed typhus fevers. As two-thirds of our readers, however, cannot now have access to the volume of our *quarterly* series in which this discussion was recorded, we shall quote the following passage from Dr. Curry's speech:—

"Here Dr. Curry took occasion to pass an eloquent, and not unmerited eulogium on our own immortal Sydenham, whom he looked upon as little inferior to Hippocrates himself. The longer Dr. Curry lived, and the more he saw, so much the more strongly was he convinced of the truth of Sydenham's opinion, that diseases obeyed a periodical influence, partly from the circumambient air, and partly from terrestrial exhalations, or from both combined. He supported this opinion by many ingenious reasons, and important facts, drawn from the histories of epidemics since the year 1793. Among other data on which he has grounded his assumptions, the following one was stated. In the autumn and winter of 1809, when the Walcheren fever produced such havoc among our troops in the Islands of Zealand, Dr. Curry affirmed, from his own personal knowledge and observation, that there was not a street or lane in this metropolis wherein remittent and even intermittent fever might not be seen among the inhabitants. Hence he concluded, that the same aerio-terrestrial influence, which showed itself so fatally in the debouches of the Scheldt, where vast bodies of men were congregated under circumstances exceedingly favourable to its full operation, was also acting with greater or less power, over England, and probably over the whole surface of the globe. It was in this way, he said, and upon this principle only, that the spread of those desolating epidemics, which in some years, ravaged the western tropics, and the countries of North America and Europe partaking of a tropical nature could be rationally accounted for. This view of the

* "Upon the whole, we think that we have been two precipitate in rejecting the opinion of Sydenham; and that no other hypothesis, if such it be, is half so plausible as the TERRESTRIAL origin of epidemic influence, however that influence may be subsequently transported about, or modified by atmospheric constitution," *Med. Chir. Rev. Dec.* 1820.

subject led him to suspect that many young men of the present day, who prided themselves on having discovered the true nature and the most effectual treatment of fever, and who supposed that their forefathers and predecessors were all in the dark, in these respects, had, in reality, but little cause of self-gratulation, since the diseases which their despised forefathers treated in this supposed unskilful manner, were very different from those of the present day; and hence that it was very probable—nay, that it was almost certain, that future generations would eye the doctrines and practices of our times with as much astonishment, and as exultingly contrast them with their own, as we do now with respect to times past. This consideration, he maintained, ought to repress the pride of the present generation."*

After having pointed out this coincidence of opinion between Dr. Curry and Dr. Armstrong, we shall now pursue the latter gentleman's statements in the paper before us.

Dr. A. observes that, 1st, the intermittent, remittent, and what is called continued typhus fevers, pass or repass into each other, as he has repeatedly observed—2dly, the remittent fever from *malaria* or *marsh effluvium*, "has a combination of symptoms exactly similar to those which occur in continued typhus fever, and which, *as a combination*, occur in no two other affections whatever." If Dr. A. therefore, was now to make a nosological arrangement, he would call the disease "intermittent, remittent, and continued typhus." In tracing back many of the cases, Dr. A. found that they had commenced as intermittents—he has seen the remittent run into the continued typhus, and the continued typhus become remittent or intermittent. Our author leaving aside, for the present, certain aberrations of the disease, which cannot easily be arranged under any systematic division of the schools, proceeds to the identification of marsh remittent and continued typhus fever, by an analysis of the symptoms which they observe in their regular and unequivocal forms. The following observations we could not abridge without loss of value in the delineation.

"The remittent form, as it occurs in this country, is always attended by a simultaneous affection of the brain, mucous membrane of the air passages, mucous membrane of the alimentary canal, and of the liver; and this peculiar combination of symptoms is accompanied by as peculiar a lassitude of mind and loss of muscular power. The affection of the brain, among other signs, is denoted by a dropping of the upper eye-lids, which, therefore, cover a larger portion of the globe of the eyes than natural; while the eye itself is more glary than in health, and yet it conveys an expression of dulness or indifference of mind, so that there is a remarkable mixture of physical brightness and intellectual muddiness in the expres-

* Med. Chir. Journal, Jan. 1819. This discussion took place in November 1818.

sion of the countenance. It is difficult to convey this mixed expression in words, but any practitioner who has once seen it could hardly mistake it again. The affection of the lining of the air passages, is partly marked by some preternaturally purplish hue of the lips, attended with more or less huskiness of the voice, more especially observable when the patient coughs; and the cough is usually slight or severe according to the degree of the affection of the lining of the respiratory passages. The affection of the mucous membrane of the bowels and of the liver, is generally marked by the evacuations from the bowels being mixed with glary mucus and dark bile, which often resembles brown melted resin. There is also frequently some obscure abdominal uneasiness on pressure, especially about the pit of the stomach. The tongue is covered by a dirty whitish fur in the centre, and its edges are usually redder than natural; but in the progress of the disease it often becomes brownish in the centre, and the breath, more particularly in cases of the continued type, has almost always a peculiarly sickly odour.

"The lassitude of mind, and the prostration of strength, are closely connected with the state of the mucous membrane of the air passages and the affection of the brain; for the lassitude and languor are always the greatest in those cases where this combined affection is the most strongly indicated. This lassitude and languor are also remarkably indicated by the voice, manner, position, and motions of the patient. The remissions, when distinct, occur under two circumstances. The patient either becomes gradually or suddenly hot; and the hot paroxysm most frequently comes on towards evening, continues through the night, and terminates commonly towards the morning, the skin at that time becoming moist and moderately warm, or quite cool, but not moist; while the pulse in both instances becomes slower, softer, and of less volume than during the hot paroxysms. Now the only difference between the remittent fever and continued typhus fever, is, that in the latter, as it appears in adults, the symptoms are more severe, and the remissions are entirely absent; the skin being hot or warm, and the pulse quicker than natural during the whole day and night, though even in that form, the pulse and heat are highest at the latter period.

"The combined affections, then, of the brain, lining of the air passages, lining of the alimentary canal, and of the liver, together with a peculiar lassitude and languor, are the true diagnostic signs of the remittent, and continued forms of typhus fever. Other parts may be and sometimes are simultaneously affected, but, if we except the spinal cord, the affections of those parts are not essential, but accidental occurrences. The continued form of typhus in particular is liable to considerable variety in its expression, as the disorder may predominate most in the brain, lining of the air passages, lining of the alimentary canal, or in the liver; but still an accurate observer cannot fail to recognise the disease from the coincidence of the above signs, though they may be slight or severe. The morbid appearances also are correspondent to the symptoms, for dissection in mortal cases shows the remains of some disorder in the circulation of the brain, lining of the air passages, and lining of the bowels, varying considerably in degree; but it is curious, that though the secretions of the liver are so generally disturbed during the progress of the disease, yet seldom any traces of organic mischief are exhibited in that organ. Certainly one of the most remarkable peculiarities of typhus, particularly under its continued form, is the affection of the mucous membrane of the bronchia; and I could show, from many facts, that it is the main cause of the varying degree of heat, of muscular and mental disturbance, and that it not only gives rise, in the advanced stages, to the peculiar dryness and darknes of the tongue, but that, it is connected intimately with those symptoms which have been termed malignant, and which the older writers found so difficult to explain on any thing like rational principles. The want of due decarbonization of the blood is the cause of many of the most remarkable symptoms attendant on typhus; but the degrees in which this process is impeded are not always proportionate to the degrees of mucus accumulated in the bronchial tubes, and spread over their lining; for in some instances the secretion on the tongue, and on the fauces, is a sort of sticky varnish, and this same secretion, seemingly, exists occasionally on the mucous membrane of the respiratory passages, when little mucus is, comparatively, accumulated there. Blood, not duly decarbonized, operates more or less as a narcotic on the brain, and tends materially to influence the animal heat and the heart's action; and hence partly arise, in the progress of strongly marked cases of typhus, the muddled state of the brain, the smothered heat of the surface, and the soft compressible pulse, which become its concomitants, however high the excitement may have been for the first three or four days." 162.

Why typhus fever assumes in one person an intermittent, and in another, the remittent or continued form, Dr. A. thinks, may probably be owing to the dose of the poison, or the condition of the recipient, or on both conjoined. Dr. A. avers that, he has ascertained, beyond a doubt, that the remittent and continued forms of typhus are complicated with internal inflammation, in one or other of the four parts above specified. This doctrine, we are sorry to observe, does not quadrate with Dr. Armstrong's former statements in respect to typhus, the simple form of which (a continued form) was said hardly ever to leave any visible alteration of structure, on dissection. We do not, indeed, deny, that some degree of excitement in some of the structures of the body takes place in most continued fevers; but we are far from believing, that it takes place coeval with the pyrexia, and still less do we consider it in the light of the cause of the febrile state. We cannot agree, therefore, with our most esteemed friend in his opinion, that "internal inflammation is probably the immediate cause why typhus puts on the remittent or continued character."* Indeed, after seeing human contagion so generally,

* Med. Intelligencer, p. 163.

we might almost say universally, (for the exceptions are not more frequent than in most other general rules in medicine,) produce the continued form of fever, and marsh miasma the remittent and intermittent forms, we cannot bring ourselves to the belief of the identity of origin in the two classes.

"The only objection, which has struck me, to this view is, that the miasma which produces the intermittent form may be originally human, and not marsh miasma, because the ill-ventilated habitations of the poor will as certainly confine the effluvium of the human body as it will marsh miasma. But after the immense body of evidence which Dr. Bancroft has collected, to shew that human effluvium, however concentrated, does not produce typhus or contagious fever, it seems much more philosophical to conclude, that it is the concentration of marsh, and not human miasma, which originally produces this disease." 163.

Dr. Bancroft has laboured hard to prove that, *dead* animal matters, however putrid, and however concentrated the effluvia from them, will not induce fever, but, neither he nor any other man has been able to prove, that the effluvium from crowded *living bodies in a state of disease*, are equally innocuous. The arguments used by Dr. Bancroft to prove that the cause of disease at the "Black Assize," and at the "Old Bailey," was marsh miasma, or a stream of cold air, are now justly considered by the profession, as mere forensic eloquence and legal quibbling to prove that black is white, and white no colour at all. In short, Dr. Armstrong himself, while he hesitates in allowing typhus fever of the intermittent and remittent forms, to possess the power of propagating itself from one individual to another —in other words, to be contagious, admits that "he has met with cases where the *continued* form of the disease, in which the secretions are the foulest, certainly appeared to propagate itself by contagion."* Now we think that, where two forms of disease have such a remarkable distinction that one is contagious and the other non-contagious, there is some ground for supposing they arise from different origins. Among other instances which our excellent and candid author could adduce in favour of continued typhus being contagious, he states the following remarkable fact.

"A very respectable woman, who performed the office of a nurse to some patients labouring under typhus, was assisting one of them from the night chair, and she became sick at the stomach, and faintish, from the offensive odour of the evacuation which he had just passed from the bowels. From that time she drooped, and a few days afterwards had a severe attack of continued typhus, characterized by its peculiar combination of symptoms. This is a striking instance, and I have met with some others which were equally, or even more striking."† 166.

---

* Med. Intelligencer, p. 165.

† For a very remarkable fact of this kind, we refer to Dr. Proudfoot's

We entirely agree with this distinguished and observant physician, that, in all probability, the contagious or non-contagious nature of typhus is dependent, partly on the quantity or concentration of the effluvium thrown off from the body, and partly upon the closeness or openness of the patient's apartment. Or we should prefer saying that typhus fever is in its nature contagious, but that the chance of its propagation from individual to individual is greatly dependent on the abovementioned circumstances.

We are sorry that the limits of a periscope will not permit us to present a full analysis of Dr. Armstrong's ingenious paper. We recommend a careful perusal of the original to our readers.

While we cannot bring ourselves to coincide entirely with Dr. Armstrong on certain etiological points, we differ from him with great diffidence, because we hold his talents and character in the highest respect and esteem.

---

While on the subject of typhus, we may allude to a curious account of a "sporadic abdominal typhus," described by our young friend, Dr. Autenrieth of Vienna, in a late No. of the Edinburgh Journal. This disease, which he thinks has not been noticed by authors, chiefly attacks people in the higher classes of life, and generally occurs either during, or shortly after the years of puberty. The symptoms are nearly the same as in the low and insidious forms of common or contagious typhus; but it arises without contagion, and the seat of the complaint is the nervous system of the abdomen rather than the brain. "A vomiting, followed by a dull pain between the stomach and navel, is quite characteristic," of this abdominal typhus. From the minute detail of symptoms, the disease does not appear to happen often in this country. In Dr. Autenrieth's own case, the complaint lasted three months. In the dissection of patients who die during the abdominal stage of the fever, that is, from the 11th to the 17th day, "there is found a peculiar inflammatory state of the nerves, principally those of the abdomen." An exudation of reddish serum was observed along the fasciculi of nerves, and the medullary substance itself was penetrated by a dark-coloured cruor. The stomach and intestinal canal, at the same time, exhibited rose-coloured inflamed spots. If the patient dies at a later stage of the disease, the usual appearances seen in common contagious typhus, will be then observed. If the above phenomena, especially as regards the abdo-

---

paper in the 72d No. of the Ed. Journal, p. 385. An athletic soldier caught fever, in Ireland, but the source of the disease could not be ascertained. While bleeding the patient one day, he breathed full in our author's face, and the impression was particularly disagreeable. He predicted that in eight or ten days he would have fever. He was seized on the ninth day, and nearly died of the fever. The soldier's wife came to see her husband, and was seized on the tenth day from her arrival. Five other soldiers, who occasionally visited this man, caught the fever. We think these are pretty authentic proofs of the existence of contagion.—*Rev.*

minal nerves, be correct, and we know Dr. Autenrieth to be a young gentleman of great probity and talent, then we must cultivate neurotomy more than we have done, while prosecuting our pathological researches.

---

2. *Reunion of entirely separated Bone.** The statements of Balfour and others leave no doubt, we imagine, in the minds of medical men, that soft parts, and soft parts containing bone, have been reunited after complete separation from the body; but we do not remember ever to have read of the reunion of denuded and detached bones. The present instance, therefore, is curious, and may be useful both in a physiological and surgical point of view.

Professor Walther having trepanned a dog and returned the disc of bone to its place, found that it united with the corresponding edges of cranium. Soon after this, a mason, addicted to spirituous liquors, received a blow, by a stone, on the head, which produced moderate symptoms of concussion, that were treated in the usual way. The accident, however, was succeeded by such pains in the head, as induced the man, after trying various remedies, to beg for the operation of the trephine to be performed. Professor W. consented, and a disc of bone was removed, but the dura mater was found healthy. The bone, after being deprived of its periosteum, was returned, and the integuments drawn over it. The febrile symptoms were moderate, and the signs of meningeal inflammation by no means severe. Reunion of the integuments did not take place—suppuration ensued—the discharge continued some months—and the patient found himself better of his head-aches, which gradually diminished, and ultimately disappeared. At the bottom of the wound the osseous disc could be felt loose. Professor W. thinking, at the end of the third month, that it ought to be removed, seized it with the forceps, but was surprized to find that, instead of bringing away the bone entire, a thin, angular, and ragged portion, consisting only of part of the external table, was removed. The inferior surface of this portion was rough and unequal—one of its margins round, the other pointed and serrated—in a word, the vitreous table of the separated disc, and a part of the external lamella, were reunited; while the larger portion of the latter was exfoliated. On attentive examination of the bottom of the wound by the probe, "the original parietal opening was found thoroughly closed, and filled by osseous matter, hard, and covered by healthy granulations." Professor W. justly concludes that, as exfoliation and granulation could only take place in a part where there was an active state of its vessels, so it was evident that the reunited portion of bone retained its vitality, and, in this case, formed vascular connexions with the dura mater, and with the diploe, thus becoming subject to the usual processes of nutrition and vascular action. The wound gradually healed.

---

* Professor Walther of Bonn. See Med. Repos. 102.

3. *Metastasis.* Among the various means by which Nature carries off noxious matters from, or prevents morbid processes in, the system, there is one, which is generally considered an evil at the time.

——— pauci dignoscere possunt vera bona.

This is a fœtid perspiration from the feet. There are many examples on record where attempts to restrain this disagreeable secretion have been followed by bad consequences. Raymond informs us, that a nun had long been afflicted with a purulent discharge from the eyelids, which, at the age of 22, changed to copious and fœtid sweats from her legs and feet. While she submitted to this inconvenience, her eyes continued strong and healthy. At length, however, these fetid perspirations became very disagreeable to herself and her associates, and, therefore, she bathed her extremities in astringent lotions, which arrested the cutaneous discharge, but brought on epilepsy, which continued, with intervals, for three years. The epilepsy now disappeared, and the patient became affected with scrophulous swellings in the neck and axilla. From these parts, there was ultimately a translation to the lungs, of which the patient died.

Reydellet records several instances of nearly a similar nature, and Dr. Thomas Harris, in an interesting paper on metastasis, published in the 17th No. of our very respected cotemporary, the American Medical Recorder, has related a case, the particulars of which, we shall here introduce to our cisatlantic readers. Dr. H. was called to a lady, in March last, affected with severe pain in the back and head, with great depression of spirits, and occasional symptoms of hysteria. She was treated by our author for nearly a month, without experiencing any relief. About this time, she informed Dr. H. that she had lately gotten rid of an old companion (as she termed it) which had been extremely offensive to herself and her friends—namely, a copious and fetid perspiration about the feet, which had succeeded a scrophulous swelling in the groin of long standing. These perspirations had continued three years, during which she enjoyed uninterrupted health, until lately, that she had caught cold by getting wet feet. Our author very judiciously took the hint, and prescribed pediluvia, the vapour bath, and emollient poultices. The fetid sweats returned, and with them, good health and spirits.

The study of metastatic affections, both in a pathological and therapeutical point of view, is not sufficiently attended to by modern practitioners. We agree with Dr. Harris that it should be laid down as a rule of practice, in every chronic disease—"to favour a metastasis to the surface." When the disease exists in the cutaneous expansion, it will be no less the duty of the practitioner to guard against a translation from that, to an internal structure.

4. *Axillary Aneurism.** Our surgical brethren are well aware how few successful cases (or even attempts) there are on record of the kind now before us. We conceive that, next to the aorta itself, the subclavian artery is the least accessible to the ligature, especially in the living subject, and where disease has deranged all the relative position of parts. Mr. Todd is entitled to the greatest praise for his intrepidity and dexterity in an operation of such tremendous difficulty and danger.

The patient (John Dundas) was 53 years of age when he entered the Richmond Hospital, on the 21st of January of the present year. He stated, that he had been uniformly healthy and temperate till early in June 1821, when he felt a stiffness and numbness in two of the fingers of the right hand, which, in a month, was attended with inability to close the fingers. The hand then, and ultimately the arm, became œdematous. In October of the same year, he perceived a small tumour in the axilla, which rapidly encreased, attended with weight, pain and pressure. He went first into the Fermanagh Infirmary, and from thence was transported to the Richmond Hospital. The aneurism, at this time, not only distended the axilla, so as to cause the scapula to project considerably backwards, but it was very prominent anteriorly—its base extending upwards to the clavicle—inwards to the edge of the sternum—downwards to the nipple of the breast—and on the side of the thorax, to the upper edge of the sixth rib. The tumour was tense, elastic, and pulsating—the skin feeling stretched upon it, but not discoloured—slight pressure did not give pain; but the patient complained of deep-seated uneasiness. The whole extremity was œdematous, and the elbow separated to a great distance from the side. No pulsation could be perceived in the radial or ulnar artery of the diseased limb, but there was little diminution of animal temperature.

On the 25th of January, the pain of the arm was so intense, and the size and pulsation of the tumour so much increased, that venesection and opiates were necessary. No permanent advantage being gained by any means pursued, it was determined to have recourse to the operation, which was performed on the 8th of February, in the presence of several surgeons of the Irish metropolis. We deem it proper to give the steps of the operation in Mr. Todd's own words.

" The patient was placed on a table, lying on his back, with the upper part of his thorax somewhat raised; his head and neck inclined to the left, and his right shoulder as much as possible depressed by an assistant steadily drawing down the arm of that side. A slightly curved incision was made through the common integuments across the lower part of the neck, commencing about two inches above the acromial, and terminating half an inch above and to the outer side

* A Case of unusually large Aneurism of the right Axillary Artery; in which the Subclavian Artery was tied. By Charles H. Todd, Esq. Senior Surgeon to the Richmond Surgical Hospital. Octavo, p. 13. Dublin, 1822.

of the sternal extremity of the clavicle. The convexity of this incision was downwards, so that by a little dissection of the integuments upwards, a small flap was made, which afforded ample room for the subsequent stages of the operation, and evinced the inutility of a more extensive, or a more complicated division of the skin.

" The next part of the operation consisted in dividing the platisma myoides, fascia, and subjacent cellular tissue; this occupied a considerable time, in consequence of the great number of veins which it was found necessary to secure with ligatures. The external jugular, and two or three other superficial veins were easily secured, but a series of more deeply seated veins proved extremely troublesome; one branch of these in particular poured out blood in an alarming quantity, and receded so much within the layers of the fascia, that I was at last compelled to use the needle, and to include in the ligature the portion of fascia with which the divided vein was connected.

" I feel it incumbent on me here to state, that this profuse discharge of venous blood was chiefly the consequence of the veins having been divided too near the large trunk into which they opened; the blood therefore flowed freely in a retrograde direction from the subclavian vein into them, and issued from their inferior orifices; the bleeding from their superior orifices was inconsiderable and easily controlled. To have tied these veins individually, before dividing them, would have been an undertaking both tedious and difficult to execute, for they constituted a most intricate plexus of convoluted vessels imbedded in cellular tissue and layers of fascia.

" The venous hemorrhage having been at last effectually suppressed, I proceeded to search for the omo-hyoideus muscle: so much however was the relation of parts altered by the magnitude of the tumour, and consequent elevation of the clavicle, that the portion of this muscle expected to be brought into view in this stage of the operation, was situated more than an inch below the clavicle; and it was found necessary to draw it up from its concealment, and to cut it across, that the subjacent parts might become accessible.

" Having applied my finger to the edge of the scalenus anticus, I was directed by it to the situation of the artery; but at this juncture causes of further difficulty arose, chiefly from the great depth of the wound, and the doubt which the almost total absence of pulsation in the artery naturally excited in regard to its identity. It is necessary however to observe, that this obscurity in the pulsation of the subclavian artery was by no means referrible to the debility or exhausted state of the patient, but probably depended on the vessel having been flattened upon the first rib by the degree of extension to which the aneurismal tumour in the axilla had subjected it.

" For some time I could not be convinced that the feebly pulsating vessel, to which the point of my finger was applied, was really an artery of such magnitude as the subclavian; and aware of the disappointments which others were reported to have sustained in this operation,* I resolved to satisfy myself and my assistants upon a

---

" * See Sir Astley Cooper's case in *Lon. Med. Review*, vol. II. and point of so much importance, before a ligature should be applied. The depth of the wound rendered it impossible to see to the bottom of it, accordingly, I kept the point of my left fore-finger on the vessel, and cautiously detached it from its connexions with the blunt extremity of a director; having then introduced the fore-finger of my right hand also into the wound, I succeeded in compressing the vessel between the ends of my fingers, when the pulsation of the tumour immediately ceased, returning when the pressure was discontinued. This expedient was conclusive, and, for obvious reasons, more satisfactory than that of pressing the artery downwards against the first rib.

" From the unusual degree of displacement of the clavicle, it was expected that great difficulty would have arisen in the application of the ligature to the artery; I was therefore provided with the several instruments which have been recommended to facilitate this step of the operation, however none of these were employed, as the object was speedily effected with a common aneurism needle. At first I attempted to pass the needle in front of the artery, with the view of giving every security to the vein; to this the position of the clavicle constituted an insuperable obstacle; I therefore directed the needle along the margin of the scalenus, and then insinuated the point of it under the artery from behind, guarding the vein with the fore-finger of my left hand, until the point of the needle was sufficiently elevated. I was then enabled to seize the ligature with the extremities of my fore-fingers, which I had introduced into the wound, nearly in the same manner as when compressing the artery, and the needle being held by an assistant, one end of the ligature was drawn out anteriorly, and the needle was removed.

" The artery then lay upon the ligature; and I requested that my assistants, and such other professional gentlemen as could conveniently approach the table, should convince themselves of this fact, by making the most accurate examination. The knot was now tied, and a sufficient tightness ensured by the ends of the ligature having been passed in the ordinary way through the serre-nœud. On the ligature being tightened, the pulsation of the tumour entirely subsi-

---

Dr. Rutherford's account of M. Dupuytren's unsuccessful operation at the Hotel Dieu, in *Edin. Med. and Surg. Journal*, No. 63.

" An eminent English Surgeon, then on a professional tour, was present at M. Dupuytren's operation and dissection, and favoured me with an account of them, which corresponds with that given by Dr. Rutherford. In the same letter (dated June 21, 1819,) he states that, 'three days since a surgeon of great celebrity attempted this operation, on a most admirable subject, and in an early stage of axillary aneurism, but he could not even find the vessel, and abandoned the operation.'

" Mr. Samuel Cooper informs us, that one of the cervical nerves may be mistaken for the subclavian artery, in consequence of the pulsation of this vessel being communicated to all the adjacent parts; and that he has seen a mistake of this kind actually made by very skilful surgeons. *First Lines of the Practice of Surgery, vol. I. p.* 319."

ded; its tension was considerably diminished, and the patient felt an increased degree of numbness of the arm; the external wound was then dressed, and he was laid in bed with the limb supported on a pillow by his side." 9.

The patient did not appear much exhausted, notwithstanding the loss of venous blood during the operation. At night an opiate was administered, and, for the first time, since his admission into the hospital, produced tranquil and refreshing sleep. On the following day, there were no alarming symptoms, and the tumour was obviously diminished. There was some dyspnœa towards the evening, which was relieved by venesection. These attacks of dyspnœa recurred frequently, and frequently required bleeding and digitalis, with aperient medicine. The symptomatic fever which succeeded to this painful operation, was inconsiderable. On the 14th of February the wound was dressed for the first time, and the sutures removed. On the 15th, several ligatures came away—the discharge being small and healthy. The œdema of the limb had greatly subsided—no pulsation could be distinguished in any of the arterial trunks—the temperature of the limb had continued at 96°—the pulse at 92°. We cannot pursue the diurnal reports. On the 3d of May, when the paper closes, the man's "amendment has been most satisfactory; —the tumour is still further reduced, and he is gradually regaining the power of the muscles which move the arm on the scapula. The joints of the wrist and fingers have lost much of the unnatural laxity, and the integuments of the hand no longer possess the diseased appearances reported on the 20th of March."

We need hardly say, that this operation exhibits one of the most splendid triumphs of modern over ancient surgery. It must be received as a strong testimony in favour of the expediency of tying the subclavian artery, even under circumstances of peculiar difficulty and danger.

5. *Malignant Ulcer of the Prepuce.** Mons. L. 23 years of age, came under M. Dubois's care, presenting the following symptoms: —the prepuce was much enlarged and indurated; and, on the inferior part, was a large, deep, and eroding ulcer, which penetrated to the glans penis, with rugged edges of a violet colour, and violent lancinating pains. The disease had resisted various antisyphilitic modes of treatment, including mercury taken by the mouth. The ulcer was dressed with anodyne applications, and although the patient was very weak, he was immediately put on a course of mercurial frictions. Bitters, the cinchona, and anti-scorbutics were administered internally. Between the 12th and 15th days, the salivation became established, and quickly became inordinate, his tongue, lips, and whole face being greatly swelled. The ulcer instantly put on a healthy appearance and was soon healed. The cure was complete.

* M. Dubois. Annuaire Med. Chir.

Our readers will recollect, that in our last Number, we mentioned a case of sloughing chancre arrested in its course by mercury. We have received several communications lately, corroborating the good effects of similar treatment.

6. *Oil of Turpentine.** Dr. Gibney thinks, that this medicine has not been so liberally prescribed as it deserves. In his practice, at least, it has proved as efficacious in the ascarides, vermiculares, and lumbricoides, as in the tænia. In small doses, he thinks, the effects of the medicine will probably be felt, where they are least wished—in the kidneys or skin, for instance. A small quantity will rarely act on the bowels, and there is not so much to be dreaded from a large dose as people imagine. "There are few children of three years of age, who will not bear from one to three drachms, given at intervals." To those more advanced in years, we may order from three to six drachms. To adults, of course, it may be given on a still more extensive scale. It should, Dr. G. observes, be given as little combined with other medicines as possible, and always on an empty stomach, in doses repeated at short intervals. Our author's method is, to order a good dose early in the morning, and repeat it every hour for three or four hours, as the strength of the patient, or the presence of the disease may indicate. Although food of any kind should be prohibited at the time, yet it may be necessary to allow the patient to drink warm tea, or acidulated barley water, to allay his thirst. Should the medicine prove slow in its operation, a little castor oil, taken a few hours after, may be serviceable. Our author mixes the oil of turpentine with some mucilage, cinnamon water, and syrup. The addition of a few drops of some essential oil, or warm aromatic tincture will sometimes be useful. In some instances, the disease yields to a single dose of the remedy, and the stools assume a natural appearance—in others, a more prolonged course is necessary. "In all cases, however, it will be advisable to continue the medicine (observing an interval of three, four, or five days between the regular doses) for some time after we have reason to suppose the worms have been destroyed." The permanent healthy appearance of the alvine discharges will, he thinks, be a tolerably faithful index upon this point. Dr. Gibney illustrates the foregoing rules by a sufficient number of appropriate cases, selected from his practice. These we need not notice here.

7. *Chronic Rheumatism.*† The datura stramonium grows very plentifully in almost every section of the United States. Dr. Z. has been in the habit of using this medicine, in the form of tincture and

* Dr. Gibney. Ed. Journal, No. 72.

† Dr. Zollickoffer. Amer. Med. Recorder, No. 17.

ointment, in cases of chronic rheumatism, with (he says) much advantage. His formulæ run thus:

TINCTURE.

℞. Sem: Daturæ Stram: ℥j.

Spir: Vini Tenuis Oss.

Mix and macerate for seven days, then strain, filter, and add to it the following articles:—Ol. pulegii, gt. xx. ol. cinnam. ʒss. tinct: opii, ℥j. spir. vini camph: ℥ij. This compound tincture of stramonium, he says, will be found a most valuable stimulant and rubefacient application in a variety of cases, in which camphorated spirits and other liniments are employed externally.

The ointment is made by simmering an ounce of the flowers of stramonium, with four ounces of lard, and an ounce of white wax.

It is particularly in chronic rheumatism that our author has used the tincture, exhibiting it internally, morning and evening, commencing with about eight drops, which can safely be increased two or three drops occasionally, until the patient is sensible of its producing vertigo, when the medicine is to be suspended for a time, and, if thought necessary, recommenced again. The tincture and ointment may also be externally employed. Purgatives should be exhibited during the use of these medicines.

---

8. *Congenital Dyspepsia.** The editor of one of those numerous periodicals which, in their turn, have strutted their hour upon the stage, and then retired—some with plaudits—some with hisses—but most of them in solemn silence, has again appeared to vindicate some antiquated claims in surgery and midwifery. With these we have no concern; but, an appendix is added, on the subject of what the writer denominates, "Congenital Dyspepsia," or, in other words, that idiosyncrasy of *stomach*, from which few are entirely free, and through the influence of which, we account for those antipathies to certain kinds of food, or the disagreement of certain articles with the stomach, however much they may be relished by the palate. By way of illustration, the writer first relates his own case. Till the age of twenty, he was much in the habit of using *milk*, once or twice a day, as an article of food. He was then often afflicted with acidities in the primæ viæ, pains in the stomach and bowels, flatulence, nidorous or greasy eructations, and other accompaniments of indigestion. In point of general health, however, he was very well. Experience at length taught him, that every alimentary article, into the composition of which, either milk, cheese, or egg, entered—nay, even poultry or young pigs, fed on milk and barley meal, had the effect of deranging the functions of his stomach and bowels. The idiosyncrasy ascertained, the cause of these symptoms was avoided,

---

* Medical and Surgical Spectator, additional, 1822.

and the effects altogether ceased. He can renew them—we were going to say, *at pleasure*—by taking a bit of cheese or a bason of milk, to this day.

When dyspeptic patients are otherwise in good health, our author suspects that this congenital dyspepsia or idiosyncrasy is at the bottom of the mischief; and unless the offending article be ascertained, all medicine will be unavailing.

The author next introduces the case of a medical friend, and in the Doctor's own words. Of this case, which is not devoid of interest, we shall here present an outline.

Dr. ———, ætat. 35, subject to dyspepsia, and constipation of bowels, attended at times with considerable pain in the abdomen, complained in October, 1809, of almost constant pain in the lower part of the belly, with such an increase of the constipation, that laxatives, and even the bath-water injections, with difficulty cleared the bowels of hardened scybala. He continued in this state during the whole winter, with the exception of a week or two, after taking blue pill to affection of the mouth, during which short period, he also had regular stools. The complaint, however, returned, and the pilula hydrargyri had now no effect. Various remedies were tried in succession, but no relief was obtained. This was his state in November 1810, when, at the suggestion of the writer, he altered his diet, by substituting oatmeal gruel for milk, which last he had used freely for several years. He soon became free from pain; and although the bowels did not immediately perform their office properly, a very trifling laxative medicine was necessary. He now recollected that during the interval of ease which he enjoyed in the spring, he took whey at breakfast instead of milk.

Congenital dyspepsy, our author observes, is not confined to milk, eggs, or cheese, for its cause. One person shall be rendered dyspeptic by eating corned beef, another by an apple and so forth—every individual has his peculiarity of temperament, and his gastric idiosyncrasy. We think these hints of the "Medical Spectator" not undeserving of professional attention, and we shall be glad to see another gazette extraordinary from the same source.

9. *Hernia.** According to Mr. Lizars, all that we have learnt in the dissecting room respecting inguinal and crural hernia, "is of no avail," when we come to operate. This is a melancholy reflection; but we trust Mr. Lizars's assertion is not quite correct. We know well indeed that living and dead anatomy are two very different things; but still we venture to affirm, that the knowledge gained in the dissecting room is useful in the operating room. If not, why does Mr. Lizars continue to lecture and demonstrate?

* Mr. Lizars, Ed. Journal, No. 72.

We agree with Mr. L. that, in general, when we are cutting down, in the living subject, on a strangulated hernia, we look in vain for the distinctions between fasciæ which may be demonstrated in the dissecting room—or rather we should say, that we shall find more fasciæ than we expected, and that inflammation often converts common cellular tissue into apparent laminæ of fascia. We agree with Mr. Lizars in another point, namely, the difficulty of distinguishing between crural and inguinal herniæ in women. In an early period of our professional career, we lost a sum of money by betting with a colleague that a hernia, of which an old woman died, was of the inguinal kind, when dissection shewed it to be crural. We have, since that time, seen some able surgeons puzzled, and even deceived, on the same subject. It appears, from Mr. Lizars, that, instead of advancing in the knowledge of inguinal anatomy, we are retrograding. "Work after work," he says, "has been published on the subject, and the more and more confused and intricate are the descriptions." Vesalius, Eustachius, Cowper, Albinus, Douglas, all have erred—and the anatomy of Hernia "may be pronounced the arcanum of surgery." From this disconsolate representation we gladly turn to a prospect more cheering—that of curing herniæ (reducible) in men, women, and children, without any surgical operation at all. It is well known that the skin and other textures of an animal, when dead, are hardened and strengthened by tanning—why not tan them alive? The idea has been long acted on, as far as the stomach is concerned. That organ we are daily in the habit of tanning with bark, steel, astringent wines, &c. The transition was very natural to the parts through which a hernia descends. For many years past our author has used for reducible herniæ "a strong decoction of oak bark with *wonderful* success." Now, although we do not deny that the success might be *wonderful*, yet we do not like the expression, "wonderful success," from the mouth of a regular surgeon. Be that as it may, we think the proposal, as it is perfectly harmless, is well worthy of a trial, and we freely contribute to make the measure extensively known. A few pounds of oak bark are to be macerated in a sufficient quantity of cold water, for twelve or twenty-four hours, and then the bark and solution must be put into a larger vessel, and kept at the boiling temperature over a gentle fire for two or three days, adding, when required, boiling water, from time to time, so that the bark may be always covered. The solution should be ultimately strained, and evaporated to the consistence nearly of an inspissated juice. When used it should be warmed, to suspend the astringent matter. The hernia being previously reduced, the groin is to be bathed with the decoction, and the truss applied, three or four times a day. Mr. L. has cured hernia of many years standing, in this way, in the course of a few weeks. In general, however, it requires a perseverance of three months. This invaluable remedy was first mentioned to our author by a merchant, who cured himself after having laboured under the disease for many years.

In conclusion, we are exceedingly sceptical as to the *tanning pro-*

cess on the *living tendinous* expansions composing the apertures through which herniæ protrude.

---

10. *Inguinal Hernia, attended with peculiar circumstances.** John Morize, a young lad of eighteen years, entered the MAISON DE SANTE with strangulated inguinal hernia of the right side, which had continued thus three days. When admitted, there were acute pains in the inguinal region—tension of the abdomen—violent colic—bilious and stercoraceous vomitings—hard pulse and flushed face. Such threatening symptoms demanded the operation immediately, and it was performed on the spot. As soon as the sac, which was very thin, was opened, a knuckle of intestine presented itself of a deep brown colour, and with several livid spots, some as large as the finger nail. On the right side of the knuckle of intestine was observed a round, smooth, and brown coloured protuberance, about the size of a nut. The ring being divided, the intestine was returned with the greatest care and caution; in doing which, the little tumour abovementioned burst, and discharged full two table spoonfuls of black blood, thick and fetid. Morize was now put to bed, and diluent beverage prescribed, together with emollient lavements twice a day. Nevertheless, the abdominal pains continued, together with the stercoraceous vomitings, and obstinate constipation. On the second day, in addition to the above symptoms, the pulse was feeble, the look fixed, and the features decomposed. On the third day, when every thing indicated a fatal termination, the alvine evacuations appeared—the vomitings ceased—sleep supervened—and all the bad symptoms quickly vanished.

It would appear, from the foregoing case, that the strangulated portion of intestine had suffered so much in its vital powers, and even physical structure, as to be a long time in regaining its proper functions after the stricture was removed. The case, while it offers no ground for delaying the operation beyond a reasonable period, holds out a hope that, even when at a late period, the operation may be successful, as nature has great restorative powers in reserve for emergencies like the above.

---

11. *Emetics in Constipation.* Dr. Hosack, of New York, has published some observations on this subject, which we shall briefly notice. In the year 1796, Dr. H. communicated to Dr. George Pearson of this metropolis a remarkable instance of constipation of the bowels yielding to blood-letting and the exhibition of mercury to ptyalism, after the disease had continued twenty-one days.

"Reflecting upon the manner in which mercury operates in relieving cases of this nature, after the gums have become affected, it

---

* M. Dubois. Annuaire Med. Chir.

occurred to me that the action of this metal is not only by its effects upon the secretions in general, but more especially upon the biliary organs. The dark coloured and acrid discharges that succeed to this operation of mercury, appear favourable to this explanation. In like manner, the general derangement of the digestive organs, the foul tongue, the offensive breath, the sallow complexion, that usually precede and attend upon the first stage of this disease, no less point to this obstructed and torpid state of the liver, as having great agency at least in predisposing to the disease in question, while a check of the perspiration, and certain articles of diet, are not unfrequently the exciting causes of irritation, and of the spasm and inflammation which ordinarily constitute an attack of this complaint. With these views I was led to the use of emetics, as best calculated to remove the hepatic obstruction which appears to lay the foundation of this disease, while by their febrifuge and anti-spasmodic operation they are no less useful in removing the fever, the inflammation and constriction that constitute some of the most distressing, as well as dangerous symptoms, that attend a constipated state of the belly." *Med. Repos. No.* 103, *p.* 78.

Dr. H. proceeds to relate several cases in illustration, of which we can notice but one or two in this place.

*Case* 1. Mr. W. B. had been relieved by mercury in severe constipation on a former occasion, but was now again seized with obstinate obstruction of the bowels. His countenance was sallow, his tongue furred, breath offensive, stomach disturbed, but the arterial system tranquil. An emetic of tartarized antimony operated freely, producing a general relaxation of the system, and discharging by vomiting dark coloured viscid bile, that appeared to have been long pent up. Copious evacuations by stool succeeded, and the patient was instantly relieved.

The second case was that of a merchant, who had been several times previously relieved of dangerous constipation by mercury, and who was lately visited by a severe attack of the same kind. Dr. H. found pain and violent constriction of the bowels, small pulse, sallow and livid countenance. He gave him fifteen grains of hippo and two of emetic tartar. The medicine operated freely both upwards and downwards, and the patient was immediately relieved. The other cases are nearly of a similar nature to the above, and need not here be detailed. We shall, however, introduce to our readers Dr. Hosack's conclusions from the above and other facts which he has stated.

" ' 1.—That a constipation of the bowels is naturally attended with, and frequently produced by, a torpid state of the liver, and consequent deficiency of the biliary discharge.

" ' 2.—That the pain, spasmodic constriction, and inflammation, attendant upon this disease, are the result either of the mechanical obstruction occasioned by the deficiency of bile, and, consequently, a retarded peristaltic movement of the intestines, or the effect of a sudden change of perspiration, or of a particular article of diet.

" '3.—That in the commencement of constipation, or in its more advanced stage, when the symptoms of inflammation have been subdued by the lancet, emetics may be very advantageously exhibited, both for the purpose of removing the hepatic obstruction, and of counteracting the spasmodic constriction and pain ordinarily attendant upon this disease.

" '4.—That the salutary effects which have been occasionally derived from injections of tobacco smoke are attributable to the general relaxation, the nausea, and, in some cases, the vomiting, which that narcotic produces.

" '5.—That the benefits that have, in like manner, been obtained in some cases from the use of tartarized antimony, administered by injection, are to be accounted for by the nausea and vomiting that have been the effects of its operation, but which are to be obtained with more certainty from the same medicine given by the stomach, and to the extent of full vomiting.

" 'In like manner, inasmuch as a torpid state of the liver and a diminished secretion of bile are generally known to constitute a part of the proximate cause of dysentery, we obtain a satisfactory solution of the salutary operation of injections of ipecacuanha in that disease, as advised by Dr. Thomas Clark; but which effects are more certainly to be obtained from the use of ipecacuanha given by the stomach, as prescribed by Sir John Pringle, or of tartarized antimony, as recommended by Senac.' " P. 81.

There can be no doubt that a deranged function of the liver is very often a cause of constipation as well as of several other affections of the intestinal canal; but we cannot help viewing Dr. Hosack's theory as rather too exclusive. We do not see, however, any material objection to the practice recommended by Dr. H. Indeed, we have long been convinced, and have often stated our conviction in this Journal, that the judicious and timely exhibition of emetics is too much neglected in modern practice—purgation appearing to absorb the attention of the physician almost exclusively.

---

12. *Belladonna.** Mr. Thompson has made some observations on belladonda in neuralgic affections, and related two cases in illustration. The dose of this medicine, he thinks, should seldom be less than two grains of the pure extract—and sometimes, when pain is excessive, three or four grains, repeated at intervals of five or six hours, till its effects are manifested, may be necessary. The observation of Haller, however, must be borne in mind, that permanent blindness has resulted from the use of belladonna. Its effects on the system therefore should be carefully watched. The first case related by Mr. T. was that of a lady, who became affected with excessive pain in the centre of the left tibia, which would continue

* Mr. Ed. Thompson. Med Repos. 103.

a few hours, then abate, and at length cease. Nothing was apparent in the limb, nor did pressure occasion any pain. She tried a variety of internal and external remedies without any benefit. She was desired to take two grains and a half of extract on an empty stomach. The consequent vertigo, faintings, and dimness of sight, were such as to keep her in bed all the next day. For thirty or forty hours she could not raise her head from her pillow, without experiencing a strange and disagreeable sensation. The pain of the leg left her, however, and did not afterwards return.

In the second case, a gentleman had been seized in March with racking pains in the gum of the left side, extending along the face, for which a tooth was removed, without producing any relief. He was leeched, blistered, and took opiates, without benefit, and then another tooth was extracted, but still the pain continued. Two grains of the extract of belladonna were given, and repeated. The four grains produced slight delirium, and great vertigo. These symptoms soon left him, and the pain returned no more.

---

13. *Over-Dose of Opium.** On the 2d of March, 1794, a Mogul gentleman swallowed about 150 grains of opium, and was found, in less than an hour, in a state resembling profound intoxication—his skin of a red inflamed colour—all the veins greatly distended. When roused a little, he complained of an intolerable itching all over his skin—pulse from 70 to 80, full and soft. Fifteen grains of sulphate of zinc were administered, and ten grains more every ten minutes. This produced but slight vomiting. Fifteen grains of the zinc were then given every ten minutes till full vomiting was effected. He took in all 125 grains of the sulphate. He quite recovered. Vegetable acids were given after all smell of opium disappeared from the ejected matters.

---

14. *Venereal Affection of the Heart.*† Corvisart and most of the French pathologists insist on it that the venereal virus falls occasionally on the heart, and there evinces itself particularly in the form of syphilitic excrescences, or vegetations on the valves of the organ. We have met with three cases of this kind, in middle-aged men, but where there was no evidence, at the time of their death, that they had laboured under incompletely eradicated syphilis. Still, as M. Dubois observes, the *causes* of a disease may be removed, but the *effects* may long remain. The following case is in point.

"Claude François, a jeweller by trade, aged 34 years, entered the Maison de Sante on the 18th October, 1818, presenting the

---

* Dr. A. Kennedy. Ed. Journal, No. 72.

† M. Dubois. Annuaire Med. Chirurg.

following phenomena:—moderate pyrexia—cerebral functions undisturbed—respiration laborious—abdomen somewhat tumid—pulse irregular—tongue white—countenance pallid—lower extremities œdematous—skin of the forehead, chest, and back covered with a pustular and crustaceous eruption, which, in our author's opinion, indicated the existence of syphilitic virus in the system. The state of the patient forbade the use of mercurials, and therefore only aperient drinks were prescribed. The breathing now became hourly more and more difficult—the patient could not lie down—the thorax, on percussion, emitted a sound indicative of effusion—the heart beat over a great extent of surface, which indicated lesion of that organ. He died on the ninth day after entering the hospital.

*Dissection.* Both cavities of the chest contained serous effusion—heart considerably enlarged—parietes of the left ventricle thrice their natural thickness, but soft and flabby. Both the mitral and tricuspid valves were fringed with numerous venereal vegetations, of different sizes. Auricles natural.

When, to the more common symptoms accompanying organic affections of the heart, we have added irregularities in the pulse, we have always found it a very unfavourable omen, as it almost invariably indicates *valvular* disease—the worst species of cardiac lesion. We were lately in attendance on a dreadful case of this kind, with Dr. Farre, Dr. Walshman, and Mr. Browne, a very able practitioner of Camberwell. The symptoms were such as above described, with the addition of a tremendous regurgitation into the internal jugular veins, which pulsated like immense arteries. The struggle of the patient with this fatal disease was long, and afflicting to himself and friends. At length he sunk from hydrothorax, and serous effusions in all parts of the body.

15. *Tobacco in Phlegmasiæ.** Dr. Page has related three cases of phlegmasiæ, where nicotiana in glyster was employed as an auxiliary; but it is only on the first case we shall animadvert. A woman of plethoric habit was seized with pneumonia, and after bleeding, blisters, and a dose of salts, she has an opiate draught given her at night, notwithstanding great dyspnœa. This practice was persisted in, and we have no hesitation in saying it was bad practice. Antimony should have been given, as the grand auxiliary to venesection, and this would have done all that the tobacco glysters effected, in a far more safe and manageable manner.

16. *Flux of Blood from Hepatic Enlargement.*† Dubuc, a

* Dr. Page. Ed. Journal, No. 72.

† M. Husson. Annuaire Med. Chirurg.

watch-maker, 45 years of age, of pale complexion, but formerly in good health, had had a fall on the right side against a hard substance, since which time (three years ago) he has felt occasional pain in the region of the liver. He entered the Hotel Dieu on the 26th August, 1815, complaining of pain in the abdomen, the liver being evidently enlarged, and projecting beyond the false ribs. There was constipation of the bowels, dryness of the skin; but no fever. Leeches were several times applied to the anus, with diluents—sedatives—warm baths. On the 31st August, the patient began to pass considerable quantities of blood by stool, of a black colour, and mixed with some fœcal matters of stercoraceous smell. This evacuation rendered the abdomen less painful, as well as the immediate region of the liver, which became less tense and prominent.* These favourable symptoms led to the further application of leeches to the fundament, with diluent drinks, and emollient lavements. The discharge of blood continued about a week, when it ceased; the patient experiencing pains in the region of the liver only occasionally. The constipation of bowels obliged him to have recourse to lavements twice or thrice a day, in default of which the abdomen swelled, and the right hypochondrium became painful. By degrees the patient regained his strength—the constipation of bowels gave way—the stools became natural—the complexion more healthy. By the end of September the patient was discharged cured.

We shall take frequent opportunities of impressing on the minds of our brethren in this country the necessity of paying more attention to anal leeching, and the introduction of enemas in their practice. We are quite convinced, that in neglecting these measures we deprive ourselves of powerful auxiliaries in the treatment of diseases.

---

17. *Luxation of the Hip-Joint.*† Dislocation of the larger joints occasionally happen in places where we have but slender assistance, and simple mechanical means of reduction. It is therefore well to be acquainted with those instances where the reduction has been effected without the aid of machinery, as in the following case. On the 28th of January, 1822, a soldier, 38 years of age, of very robust habit, was violently run against and upset by a stage coach, in turning one of the streets of Paris. He was with difficulty got into a cabriolet and conveyed home, where M. Devilliers immediately saw him, and recognized a luxation of the thigh bone, by the

---

* This verifies the ancient medical precept in Serenus Samonicus.
" Si modicus pleno manat de corpore sanguis
" Subvenit:—at nimius cum vita funditur ipsa."
*Serenus Samon. de Med. Præcept.*

† M. Devilliers. Journ. Gen. de Medecine, Fevrier, 1822.

following signs, viz. remarkable shortening of the member—rotation of the toes outwards—the knee abducted beyond the median line—a tumour in the right groin near the pubis—a depression in the situation of the cotyloid cavity—inability to place the limb in its natural posture. These signs indicated a dislocation upwards and inwards of the head of the femur.

At this time there was only one person in the house capable of rendering the surgeon any assistance, and the latter was loth to lose time by sending for assistance elsewhere. Causing this person then to take a firm hold of the patient's foot, he bent the knee, and grasping the ham with his right hand, he placed his left on the site of the tumour in the groin. Thus situated he gave the thigh bone (that is the knee) a sudden rotatory movement inwards, upwards, and forwards, while counter-pressure was made on the tumour, or opposite extremity of the femur. The head of the bone instantly snapped into the socket with a considerable noise, without much difficulty, or pain to the patient. Repose, cold applications, leeches, and the usual routine of treatment completed the recovery.

18. *Introduction of Foreign Matters into the Blood.** M. Majendie pursues his physiological career with great eclat, and his contributors are daily increasing, both in number and respectability. The physiological Journal before us bids fair to have a most extended circulation, and we shall, in future, pay our respects to its contents more frequently than formerly.

It has been observed, in all ages and countries, that the introduction of putrid and unwholesome substances into the system, as food, has been generally followed by fevers and other malignant diseases, as the histories of famines too fully prove. The experiments which have been undertaken by Dr. Gaspard, may, it is to be hoped, throw some light upon the subject; and are therefore deserving of being widely known. The first series of experiments, ten in number, consisted in the introduction of purulent matter (generally a little diluted with water) from common ulcers, into the veins and certain cavities of the bodies of dogs. Before giving the conclusions to be drawn from these experiments, we shall sketch a few of the experiments themselves.

*Exp.* I. Into the jugular vein of a dog two drachms of diluted pus were injected, The animal, at the moment of injection, became agitated, and went through the action of vomiting. He whined, appeared weak, and vomited more than six times in the course of the day. An hour after the experiment there was an evacuation of

* Memoire Physiologique sur les Maladies Purulentes et Putrides, sur la Vaccine, &c. Par G. Gaspard, M. D. Journal de Physiologie, par F. Majendie, Membre de l'Institute, &c. January, 1822.

excrements, and of thick, troubled urine, which gave some relief. Towards evening, however, he lay sick on the ground, with his legs stretched out, the respiration insensible, and the pulse weak. Ten hours after the experiment he passed blackish, liquid, and extremely fetid stools, which brought about relief, ending in recovery. Next day the dog was well. Two days afterwards three drachms of pus were injected into the other jugular of the same dog. The symptoms were in a more violent degree, and death supervened within the 24 hours. Dissection displayed no alteration in any organs. Similar experiments were again made on a dog, one on the 15th, the other on the 18th September. In the first the dog recovered, after frequent evacuations—in the second experiment he died, and on dissection the inferior portions of the lobes of the lungs were inflamed and nearly hepatized. In another experiment, where three drachms of pus were injected, the animal died in great agony of tormina and tenesmus, at the end of five hours. On dissection, the intestines appeared thickened externally—their mucous membrane was inflamed and swollen, especially in the colon and rectum.

In an experiment made on the 21st of September, where half an ounce of pus rather older and more putrid than in the former experiment, was injected, frightful nervous symptoms ensued, as wandering vision, excited sensibility, convulsions, hiccup, staggering, furious delirium, burning thirst, dyspnœa, palpitation, death in two hours, amid dreadful convulsions. On dissection, while the body was yet warm, the venous blood was found very coagulable—the pericardium contained a little effused serum—the left ventricle of the heart was thickened and inflamed, presenting, on its inner surface, spots of the colour of wine lees—the other organs healthy.

In two experiments, where pus was introduced by the serous membrane of the testicle into the abdomen of dogs, without producing violent pain at first, there soon came on vomiting, evacuation of urine, fever, and dyspnœa. In three hours the abdomen was convulsed, drawn in, and very painful on pressure. Death ensued in twelve hours. In the first dog the peritoneum was found reddened and rather inflamed, with about an ounce of bloody inodorous serum. The mucous membrane of the intestines was rather red and inflamed. In the second dog, the peritoneum was inflamed, and contained a small glassful of sanguineous serum, of a fetid odour. The mucous coat was a little inflamed. Introductions of pus into the cavity of the pleura produced nearly similar effects. When introduced into the cellular texture, it was not absorbed and only gave rise to a hard inflammatory tumour ending in suppuration.

From the ten foregoing experiments our author thinks he may legitimately draw the following conclusions.

1st. That pus introduced, in small quantities into the circulation causes considerable derangement of function, from which, however, the animal recovers, after the offending matter has been expelled from the system by means of a critical excretion of urine, or of fœcal discharges.

2d. That when it is introduced at successive periods into the same animal, death is the result.

3d. That when injected into the veins, in a large dose, it produces severe inflammation, namely peripneumony, carditis, dysentery, &c.

4th. That it appears susceptible of being absorbed, causing inflammation of the serous membrane and cellular tissue, with which it had been in contact.

5th. That the majority of symptoms occurring in hectic fevers, appear to admit of being referred to the presence of pus in the circulation.

Our author is also inclined to infer that, the phenomena exhibited in the course of cancerous ulceration, old dropsies, mercurial ptyalism, gangrenous affections, drunkenness, &c. may be attributed to the absorption of a portion of morbid matters into the blood. He thinks, that absorption frequently takes place in various diseases characterized by suppuration, either through the medium of the veins, or of the absorbents, or of both—as will be made more probable by experiments presently to be detailed.

In the 11th experiment, the author threw into the jugular vein of a young sheep, an ounce of cold water, in which had been dissolved six vaccine crusts recently taken from the arms of children, to which was added, a large drop of vaccine matter from an eight day vesicle. On the introduction of this liquid, the animal went through the action of swallowing, but without evincing pain. The animal suffered no inconvenience, and had no cutaneous eruption afterwards.

A similar experiment was performed on a little bitch, four months old, and which had not had the "maladie de chiens." Half an ounce only, of the vaccine solution was thrown in. In an hour the animal refused food and vomited. The vomiting was renewed many times, accompanied with burning thirst, evacuations of urine; and uneasiness which lasted all the day. Next day, however, all was well, and no eruption appeared.

In two succeeding experiments, putrid sanies was thrown into the jugular veins of dogs. In the first, the animal went through the act of swallowing during the injection, and soon after had dyspnœa, uneasiness, and faintness. It lay on its side, refused food, and soon after passed excrements and urine. In an hour, there came on prostration of strength, dysenteric stools, redness of the conjunctiva—pain on pressure of the chest and belly, bilious and bloody vomiting—death at the end of three hours.

On *dissection,* the lungs were found inflamed in a peculiar manner, or rather congested. They had a violet colour, and on them, as well as on the left ventricle of the heart, the spleen, the mesenteric glands, gall-bladder, and subcutaneous cellular membrane, were seen ecchymosed spots or petechiæ. The peritoneum contained some spoonfuls of reddish serum. The mucous membrane of the stomach was somewhat inflamed—that of the intestines greatly so, with black spots, and a bloody gelatinous covering, like wine lees, or the washings of meat. In the second dog, the phenomena and post mortem lesions were as nearly as possible the same.

In an experiment where a fœtid liquid, produced from the putrefaction of cabbage leaves in warm water, was injected into the jugular of a middle-sized dog, copious stools of a liquid, fetid, and soot-coloured appearance, analogous to the dejections in melena, supervened in the course of nine hours, accompanied by vomiting and great prostration of strength. The dog lived three or four days with nearly similar symptoms, and then sank.

On dissection, slight phlogosis was observed in the lining membrane of the bronchiæ—the left ventricle of the heart presented several brown ecchymoses—a fibro-albuminous concretion, weighing two drachms and a half, filled in part the right ventricle of the heart, and adhered to its internal surface by a space not larger than the finger nail. The mucous membrane of the intestines, especially of the duodenum and rectum, was inflamed in longitudinal streaks, but without any thickening or ulceration.

In a similar experiment, where a putrified solution of the stalks and leaves of beetroot was used, nearly the same symptoms as above were produced, especially dysenteric affection, with bilious vomitings, &c. The *post mortem* appearances were nearly the same. These two experiments, M. Gaspard thinks, prove that putrid vegetable matters act upon the system in the same manner as animal, but in a milder degree.

The next suite of experiments is on the subject of *absorption*. Where putrid vegetable liquor was thrown into the subcutaneous cellular tissue, inflammation arose, attended with pretty severe constitutional symptoms, but death did not ensue. Where a similar liquor was injected into the cavity of the peritoneum, severe constitutional symptoms immediately took place, such as vomiting, purging, abdominal tenderness, dyspnœa, tenesmus, and death, in nine or ten hours from the injection of the fluid. On dissection a quantity of vinous coloured, but inodorous effusion was found in the belly—the peritoneum inflamed—and, as it were, ecchymosed—the mucous membrane of the digestive canal, from the cardia to the anus, uniformly inflamed—the cavity of the pleura containing red and bloody serum. These experiments prove, M. Gaspard thinks, that putrid substances are *absorbed* when thrown into the cellular tissues, grounding this assumption on the following circumstances:—1st. The similarity of symptoms resulting from injection into the veins and into the cavity of the peritoneum. 2d. Although the injection into the latter produces violent local symptoms, still derangements supervene in different and remote organs, which can only be explained (he thinks) on the principle of absorption. 3d. The copious secretion of urine which always followed the injection, must, he imagines, arise from the absorption of the irritating liquid. 4th. The absence of the peculiar odour of the injected substance in the fluid found in the peritoneum after death. 5th. The derangements found in the lungs, &c.

Although we have no doubt of absorption in general, yet we think the author's arguments in proof thereof are far from satisfactory. The disorders induced in the system at large, and even in remote structures of the body from an acrid substance thrown on, and producing inflammation in, the peritoneum, might be accounted for, we imagine, without the actual absorption of foreign matters, on the well-known principle of the sympathy which exists between the different structures of the body.

M. Gaspard goes on to remark that the general effects of the introduction of the putrid matters in question, whether into the great cavities or the veins, appear to be a peculiar kind of inflammation accompanied with a species of passive hæmorrhage from the mucous membrane of the intestinal canal. The remaining experiments of M. Gaspard we do not consider as particularly interesting or worthy of translation to our pages at present. We leave our physiological readers to form their own conjectures, and draw their own deductions from those we have above detailed.

19. *Carcinomatous Tumour in the Neck, mistaken for Aneurism of the Carotid Artery.* A gentleman in Geneva had a tumour, which grew under the angle of the jaw on the left side, accompanied by great pain. At length it so obstructed his swallowing, that he was on the point of dying from inanition. In consultation it was agreed that there was reason to believe the tumour to be aneurismal, founded on the following circumstance. When the patient was placed in such a position that the outline of the tumour could be defined, and compared as to its size, at different periods, it was perceived that pressure on the carotid artery manifestly and considerably diminished its volume. This fact, and a consideration that the patient had but a few days to live, if not relieved, determined M. Munoir to pass a ligature round the carotid artery.*

The operation was accordingly performed with the temporary ligature. The tumour quickly shrunk, the gentleman was able to swallow, and in a short time it was reduced to one-third of its original size, with an entire cessation of the excruciating pain. When the patient and friends were congratulating themselves on the prospect of complete recovery, the volume of the tumour began gradually to augment—the pain to increase—and, in short, the whole of the bad symptoms to return. The surgeons of Geneva knew not how to proceed, and the patient was recommended to go to Paris.

* In a conversation which Dr. Marcet had with the celebrated Scarpa at Pavia, the latter gentleman observed that he had repeated all Mr. Travers's experiments on temporary ligature of arteries, both in man and brutes, and found them perfectly correct. He was therefore not a little surprized to find Mr. Travers subsequently invalidating his own doctrines, the truth of which had thus been proved by a foreign and unbiassed experimentalist.

There he consulted Dupuytren, who, with his accustomed ***liberality***, reproached the surgeons of Geneva, for having completely mistaken the case, and performed an unnecessary operation. He, of course, proposed and executed extirpation. For a time, things wore a flattering aspect again, and Dupuytren plumed himself on his penetration and dexterity; but presently the disease began to shew itself once more, and in despite of all the caustics and cauteries which Dupuytren could apply, it progressively got worse, and, in fact, assumed all the characters of a malignant or carcinomatous affection. The disease has spread not only to the skull, but to the brain, with a loss of intellectual faculties, and a state of dreadful sufferings, so that his existence is become loathsome and horrible in the extreme. Fortunately it is near a close.

Now, contrary to the fiat of M. Dupuytren, we are of opinion that the operation resorted to by the Geneva surgeons was *justifiable* under the then existing circumstances. It cut off the supply of blood from a tumour which had so far encroached on the œsophagus as to threaten speedy death for want of food—and the event proved that it did give a temporary relief, not only checking the growth, but actually diminishing the volume of the tumour to one-third of its greatest size. This operation also gave a chance for the sloughing process, (which sometimes oocurs,) by reducing the vascularity, and consequently the vitality of the morbid structure.

On the other hand, we conceive that M. Dupuytren's operation was *unjustifiable* at the time it was performed;—and for this reason, that the disease was, long before, become constitutional. Those most experienced in carcinomatous affections are aware that the period for extirpation is often too late, long prior to the scirrhus becoming an open cancer—and that even in the female mamma, where there are no important parts underneath to prevent the surgeon from searching freely for all roots of the disorder. But in the neck, where the bands or radii of the scirrhous structure shoot in among vessels and nerves where the knife dare not follow, what chance have we, at any thing like an advanced period of the complaint, of extirpating root and branch of this malignant disease? M. Dupuytren may say, "you judge of my operation by the event." We reply that we do not judge of *it* entirely by the event, but by the event, in many other cases—the safest way surely of forming an opinion in medical matters. And there can be little doubt that M. Dupuytren availed himself of the knowledge of the event, when he censured so freely the first operation on the patient by the Geneva surgeons—and when he boldly pronounced that it never had been aneurism *after* the operation proved that part of the business beyond all doubt. We freely accord to M. Dupuytren the merit of being, probably, the first operating surgeon in France; but we will not accord to him a merit of equal importance, that of knowing better than any of his brethren, *when* an operation is proper, and *when* unjustifiable.

20. *Malaria.** The learned but somewhat visionary author of the article "Malaria," in the last number of the Edinburgh Review, has made, or thinks he has made, three notable discoveries. The first is, that St. James's Park "is a perpetual source of malaria," producing intermittents, dysenteries, liver complaints, sciatica, toothache, rheumatism, neuralgia, and "various derangements of health, in *all* the inhabitants who are subject to its influence." This is glorious news for the doctors, and we expect to see a fresh colony emigrate from the East, and settle "within the verge of the palaces," as the poor's rates express it. This is clearly the reason why—

> The College is deserting Warwick Lane,†
> "For Burnham Wood has come to Dunsinane."

We always gave the doctors, as well as the lawyers, credit for keen olfactories in smelling out a suit; but really we had no idea that their sagacity was such as to discover Pandora's Box between Buckingham Palace and the Horse Guards.

The second notable discovery of our author, is that of a "miasmometer," or test for malaria, in the person of "an officer who had suffered at Walcheren," who always has a return of his ague fits if he walks up St. James's Street, or down the right hand side of the Strand! We are rather at a loss to discover the reason why the Park malaria "reaches all through Finsbury division and Whitechapel," together with the *South* side of the Strand, while the *North* side of the same street is proof against this morbific agent! It appears too that the walking "miasmometer" has detected this *courtly fiend* in *Bridge Street*, Blackfriars. Surely Alderman Waithman, of that street, will bring this into Parliament as a charge against ministers, and as a specimen of the *corrupt influence of crown lands*, as well as of crowned heads.

The third or grand discovery of our author, "will form," he says, "one of the most valuable discoveries which modern times have added to our knowledge of prophylactic remedies." This is neither more nor less than a "*gauze veil*," by which the London Peripatetic may defend himself at once from flies and miasmata, while perambulating St. James's Street, Whitechapel, the Finsbury division, and even the South side of the Strand!

The discoverer of such a valuable prophylactic would certainly be entitled to a parliamentary reward, were it not for the unlucky circumstance that Rigaud de Lille proposed the same defence against malaria many years ago, as is well known to every medical man in the British dominions.‡

In conclusion, we beg to say that the author of the paper before us cannot attach more importance to malaria than we do; for we

* Edinburgh Review, No. LXXII. Vol. 36.

† See our last number.

‡ See a translation of Rigaud de Lille's Memoir, in the *second* edition, of Dr. Johnson's Influence of Tropical Climates.

have felt its influence, and seen its effects, on a much larger scale than ever came within his observation. But living, as we have been, in the very centre of this supposed malaria of London, for years past, without seeing an atom of foundation for the hypothesis (for such we deem it) which is here brought forward, we can hardly be accused of unreasonable scepticism on the subject. It is not from such a piece of water as that in St. James's Park, and under a sky like ours, that febrific miasms are wont to arise. And even if they did, it is not upon the well fed, well clothed, and well housed inhabitants in the neighbourhood that they could make any impression. No wonder, then, that we could hardly restrain a smile, when we perused this serio-comic hoax of the sly Northern Critic on his credulous brother Bull of the South.

---

21. *Traumatic Tetanus.** Dr. Kennedy, an able and zealous cultivator of medical science, has detailed, in the 101st number of the Medical Repository, a curious case of traumatic tetanus succeeding a lacerated finger. On dissection, there were traces of vascular excitement on the peritoneal covering of the stomach—its mucous coat was preternaturally soft, and overspread with a firm yellow exudation, emitting a nauseabund odour—sphacelus obtained near the pylorus, and in the superior half of the duodenum. The liver was extensively disorganized—its substance being much softened and untenaceous, its vessels gorged with purple blood. The spleen crumbled into pieces when pressed between the fingers. The pericardium was dark, approaching a livid hue, easily lacerable, and containing two ounces of a dark brown sero-sanguiform fluid, of a syrupy consistence. The posterior hemisphere of the heart was marbled with streaks of dingy white—the anterior was nearly black. The left auricle and ventricle were unaltered. The right, with their valves, muscles, and tendons, " were so dissolved and imblended with coagula of venous blood, as to form an inorganic mass, black like pitch, and not firmer than a dense fibrinous clot." We regret that there is no dissection of the brain and spinal marrow.

In the succeeding number of the Medical Repository, Dr. Kennedy returns to the subject of tetanus, and presents us with a sketch of the principal modes of treatment which have been employed in this very formidable disease. We cannot analyze this part of Dr. Kennedy's excellent paper, but may merely observe that he appears to lean to the depletive treatment, as that which offers most chance of success. The concluding case related in the very valuable communication we shall introduce here, somewhat abbreviated.

On the 1st January, 1822, Dr. K. was summoned to a young woman who had fallen down in a fit, and was apparently dying. He found her labouring under an exquisite paroxysm of hysteria,

---

* Dr. James Kennedy, of Glasgow. Med. Repos. 101-2.

lying insensible, with the lower jaw immoveable, the cervical muscles rigid, the countenance flushed, the temporal arteries throbbing violently, the opake cornea suffused, with other symptoms indicative of sanguineous compression of the brain. About thirteen months previously she had been thrown into a similar fit by a sudden fright, since which time her catameniæ became irregular, and for the last six months suppressed. Three days preceding the present attack she had been severely struck on the lumbar region, succeeded by lancinating pain, head-ache, and catchings in the chest. Twenty ounces of blood were immediately abstracted, with the effect of awakening consciousness, and giving relief to the organs of deglutition. Half a drachm of the sulphate of zinc was then exhibited, which produced instant vomiting, and the expulsion of much crude aliment from the stomach, which afforded much relief. She took an anodyne draught, and composed herself to rest. Next day, our author found her in a state of great exhaustion, with feeble, intermitting pulse, loaded tongue, and confined bowels. An active lavement administered, and a powerful antispasmodic mixture prescribed. At six of the same day, the hysterical paroxysm returned, combined with true trismal and opisthotonic spasms. These subsided, and left her in still greater weakness than before.

"Recognizing a complication of tetanus with the original disease, I instituted a minute examination of the patient's upper and lower extremities; and, with considerable alarm, discovered a small, dry, orange-coloured ulcer on the last joint of the great toe of her left foot. This, I was afterwards told, succeeded a bruise of the parts with a stone. Repetition of the lavement and other remedies, with an emollient poultice to the ulcer, being enjoined, the assistance of my friend, Dr. Nimmo, was requested; and we met, by appointment, at eight o'clock in the evening.

"At this time, we had an opportunity of witnessing a most formidable paroxysm of the malady, by which we apprehended the misfortune of seeing our patient irrecoverably destroyed. It was a paroxysm of genuine opisthotonic tetanus, superadded to hysterical convulsions of the severest kind.

"Being satisfied of the disease's true nature, we directed the application of leeches to the lumbar spine, abdominal fomentations, stimulating cataplasms to the toe, and an alcoholic solution of croton oil, conjoined with the antispasmodic mixture, to be given in divided doses, at such intervals as the state of deglutition should admit. The primary results of this treatment were indeterminate; the ultimate completely favourable.

"Various modifications of these remedies, aided by occasional injections and sponging of the surface with warm water, were assiduously employed during the ten subsequent days; by which time, all the symptoms, except a general nervousness, had altogether disappeared."—*Med. Repos.* 454.

So soon as general suppuration was established in the ulcer, the spasmodic paroxysms gradually diminished; hence it is not unreasonable to conclude that the tetanic phenomena were caused by the wounded toe. "By the evidence of this pathological fact, our opinion of the advantages derivable, in tetanus, from judicious depletion, vascular and alvine, was practically illustrated and confirmed."

22. *Over-dose of Digitalis.** A man labouring under asthma swallowed by mistake, or rather through despair, an ounce of the tincture of digitalis, which was followed by a sound and refreshing sleep of several hours, and ultimately produced only a variation and irregularity of the pulse, which symptoms were easily dissipated by ammonia, ether, and light cordials. Dr. Fogo seems to anticipate that from this instance it will *perhaps* be found that "this drug may be given in quantities almost beyond human credibility, and with the happiest effect." Now we would just warn our younger brethren against trying experiments with large doses of digitalis, for we have seen enough, even from moderate doses, to convince us that it is a most potent and unmanageable medicine in many constitutions, and highly dangerous, if not watched with great caution. In the present case we have not the smallest doubt that the tincture was next to useless. We have had tincture of digitalis, and that from places of great note, which had no more operative virtue than proof spirit. But even if the tincture were good in this case, there was an idiosyncrasy of constitution to resist such a dose—an idiosyncrasy we may long look for again without finding it.

23. *Ascites cured after repeated Tapping.*† We all know how rarely we cure ascites, after having recourse to the operation of paracentesis abdominis. Independently of this circumstance, however, the case under review is well worthy of the practitioner's attention.

On the 11th June, 1821, D. M. 24 years of age, presented himself to Dr. M'Carthy, with evident fluctuation of the abdomen, and œdematous legs. After exposure to cold a fortnight before, he was immediately attacked with the abdominal swelling. He was bled from the arm, and purged with jalap, gamboge, and calomel. He was also directed to take 20 drops of tincture of squills (a pretty large dose by the way) thrice a day. The swelling increased, and the scrotum became also effused. Paracentesis abdominis was performed, and ten quarts of fluid were evacuated. He was now ordered to rub rather better than a drachm (more than an ounce is

* Dr. Fogo Ed. Journal, No. 72.

† Dr. M'Carthy. Ed. Journal, No. 72.

stated in the Journal, but it is clearly an error) on the inside of the thighs every night. He took 20 drops of tincture of digitalis twice a day, and a scruple of gamboge thrice a day. This produced good effects. By the 28th June, the effusion had entirely disappeared from the scrotum; but the abdomen was still tumid. The pulse was natural, the urine more copious, the mouth not yet sore. On the 6th July, it was necessary to tap again. The mouth was sore. He was ordered bitter infusion with carbonate of soda—crystals of tartar—mercurial liniment to the abdomen. On the 19th July the operation repeated, and eleven quarts of water evacuated. Blue pill, squills, and digitalis, internally, thrice a day. The mouth became very sore, and ptyalism ensued. The dropsical swellings disappeared, and the young man completely recovered. This is a good case, and does credit to Dr. M'Carthy.

---

24. *Contraction of the Uterus.** In the sixth number of this series, page 438, we introduced a case of difficult parturition, occasioned by contraction of the uterus round the neck of the child. Mr. Plumbe, an intelligent surgeon in London, appears to have met with a similar case. The patient was a stout healthy woman, about 25 years of age, who had had difficult labours before. She fell in labour on the 12th of September; and, on examination, the vertex presented, at the upper part of the pelvis, the diameter of the latter, (from front to rear) being unusually short. A drachm of laudanum was exhibited to procure rest, and check the harrassing ineffectual uterine efforts. On the 14th a more minute examination was made. In the absence of pain, a hand could be introduced along the forehead or the occiput of the child—but there a difficulty was found to exist—" a contraction of the body of the uterus round the neck of the child." In this state of affairs Dr. Granville's modification of Assalini's forceps was applied, and although a firm grasp of the head was obtained no success followed. It was therefore agreed by Dr. G. and Mr. P. that the head of the child should be opened. When this operation was performed, Mr. P. had still to encounter the difficulty of drawing the fœtus through the constricted part of the uterus. This, however, was at length accomplished, though with the painful apprehension that the parietes of the uterus should suffer in dragging the shoulders of the child through the constricted part. The placenta being removed, the uterus was examined, and no appreciable injury could be detected. " A large projecting ridge was found on its inner surface, extending round the greater part of its circumference, and lessening the diameter of its cavity considerably at this point, which must have undergone considerable distention in the transit of the child's body." The woman had symptoms of uterine or peritoneal inflammation; but so far re-

* Med. Repos.

covered as to be lost sight of. She died, however, about ten days afterwards in a shivering fit.

---

25. *Cancer of the Lip.** M. Richerand proposes (founded on experience) a new mode of extirpating cancers of the lips. It is needless to observe that the common method is to cut out a triangular piece of lip including the disease, and endeavour to unite the cut edges by means of pins or sutures. There is often considerable pain in the process of keeping the divided parts in contact, especially where a large portion of lip has been removed, and after all we cannot avoid deformity in many instances. M. Richerand now removes the carcinomatous portion of lip with scissors curved on their *flat* sides, and rather short in their blades. The operation is very quickly performed in this way; but it is in the after-management that the novelty or improvement lies. Instead of bringing the parts in contact by pins or sutures, the bleeding vessels are tied, and a piece of agaric is laid on the raw surface, over which lint and a bandage are placed. On removing the dressings the third or fourth day, suppuration will be found established in the wound; and from this time the mucous membrane of the mouth and the external skin of the lip daily approximate till, in ten or twelve days, they are united in a line, with scarcely any deformity—especially if care has been taken to cut out the piece in the form of a long crescent. M. Richerand called in, to witness the success of this operation, Drs. Beclard, Ribes, Breschet, and J, Cloquet, at the St. Louis Hospital. The patient was a female, and it was necessary to remove the whole of the under lip (j'avais enlevé la totalité de la lévre inferieure) from one angle of the mouth to the other. Yet in this case the removal was scarcely perceptible in a fortnight. When the parts were first removed the aspect of the patient was hideous, and a person who had not known how far Nature could operate in the reproduction of lip, would have said that the patient would never afterwards be able to retain the saliva. In this case the lip was incised below the level of the floating portion—that is, below the angle which the mucous membrane makes in turning from the lip to go up on the gums. At every dressing the lip was seen more elevated, till at length it not only covered the gums, but rose above the level of the teeth. The patient perfectly recovered, and no deformity was the consequence.

We could not suppose that M. Richerand would deceive us, but when such men as Beclard and Breschet are witnesses of the fact, no rational doubt can be entertained.

---

* Nouveau Procedé pour l'Extirpation des Cancers aux Levres. Par M. Richerand. Annuaire Med. Chir.

26. *Poisoning by Opium.* Mr. Wray, and subsequently Dr. Copland, have related three or four cases where laudanum had been taken with the intention of suicide, and where stupor and insensibility prevailed to a considerable extent. In this state the patients could not, of course, swallow emetics; but by dashing cold water on the patients, and repeating the affusion according to circumstances, the sensibility was so far roused that remedies could be exhibited, and the cases terminated successfully.

27. *Remarkable Wound of the Brain.** The records of surgery present numerous instances of severe wounds of the brain, without loss of life. We believe, however, that the following authentic fact is without a parallel.

*Case.* On the 8th of March, 1822, a dragoon, who had been wounded in a duel, was carried to the Gros-Caillou Hospital. The point of his adversary's sabre had penetrated the skull by the *right* orbit, and under the ball of the eye. How far it had gone could only be conjectured, as the weapon had been withdrawn on the spot. Baron Larrey was present at the reception of the patient. The following were the symptoms as noted by Duponchel at the next morning's visit with Baron Larrey. Hemiplegia of the *left* side of the body—comatose disposition—immobility of the iris and dilatation of the pupil—projection of the eye-ball—difficulty of speech—perfect integrity of the intellect, as the patient answered correctly to all the questions put to him. He was bled both from the temporal artery and the arm—blisters to the back of the neck—purgatives—antimonials. These medicines produced some alleviation of the symptoms at first, but they afterwards increased in violence. The patient, however, lived sixteen days, preserving the complete use of his intellectual faculties till within a few hours of his death. When the paralysis deprived him of the power of speech, he proved by signs that he perfectly comprehended all that was said to him.

On dissection, there was observed some slight effusion on the right hemisphere, with partial adhesions of its investing membranes. The weapon had entered, as was said before, under the right eye, divided the optic nerve, and the ophthalmic artery, and completely traversed through the substance of the middle lobe of the right hemisphere, the point of the sabre penetrating into the roof of the skull above, without, however, touching the ventricle. The sabre in being withdrawn violently had brought with it into the orbit a portion of cerebral substance. It was very remarkable that there was no appearance of suppuration in any part of the track of the

---

* Observation sur une Plaie du Cerveau. Par A. Duponchel, M.D. Bulletin de la Societé d'Emulation de Paris. Mai, 1822.

weapon through the brain. The stomach was found inflamed, and some ulcerations in the mucous membrane of the intestines.

It is curious to think how a man could live sixteen days after the infliction of such an injury, and preserve his intellects till the last. This circumstance, our author thinks, is a proof of the truth of the Gall and Spurzheim doctrines, that the brain is a congeries of organs, each destined to govern a separate order of functions or faculties. Now we can see no other proof in the case before us, than that the brain, like some other organs in the body, is *double;* and that *one side*, like that of the lungs, or like one of the testicles for instance, is capable of performing the functions of *both*, in cases of necessity. The phlogosed state of the stomach, and the ulcerated condition of the lining membrane of the intestines, shew the strict sympathy which exists between the brain and these parts, and the reaction which one is capable of exercising on the other.

Baron Larrey, in his *Recueil des Memoires de Chirurgie*, observes, that he had long remarked that the lesions or alterations of parts about the base of the brain were followed by a diminution or loss of sensitive and locomotive powers, together with great disturbance in the function of respiration, while the intellectual faculties maintained their integrity. The Baron relates several cases in illustration of this point, for which we refer to the volume itself, page 198, *et seq.*

28. *Counter-Irritation.** Mr. Ogden has related, in the last number of the Medical and Physical Journal, a curious case illustrating the effects of extensive counter-irritation. The patient was a child, six years of age, who had been affected with cough and copious expectoration "from the earliest period of life." During the last two years he had also been subject to epileptic fits recurring at intervals of nine or ten weeks. But *these* were always brought on, or preceded by, a deranged state of the alimentary canal. On the 22d October, 1821, the child's clothes caught fire, and he was burnt in a dreadful manner, the skin being removed from the whole of the thorax in front, and a considerable portion of the abdomen. Unfortunately too, the terebinthinate dressings produced "a universal erythema of the skin." As a compensation for all these evils, the child was completely cured of his pectoral and epileptic complaints. Although we cannot recommend the *same remedy*, we think the case affords a strong proof of the great power of extensive cutaneous irritation and exulceration over internal disease.

*Hydrophobia.*† Thirteen persons were bitten by a rabid wolf on the 1st of November, of whom nine died, and four survived.

* London Medical and Physical Journal for August 1st, 1822.
† Professor Brera. Mem. della Società Italiana de Modena.

These four were submitted to medical treatment. The first patient was wounded in the neck, in two places. The wounds were healed by the tenth day; but on the nineteenth the cicatrices became red and painful. A warm bath—a drachm of mercurial liniment daily to be rubbed in—one of the following pills every three hours:—oxy. hyd. gr. ss. pulv. rad. belladonnæ gr. x. ft. pil. iv. These pills were taken till the 25th, the oxymuriate being increased to two grains, and the belladonna to thirty in each mass of pills. Salivation and faucial inflammation ensuing, the mercury was discontinued, and the bath and belladonna persevered in, the latter being now taken in the dose of four scruples *per diem*, and gradually increased (in the course of nineteen days) to three drachms. On the 1st December the mercurial treatment was resumed, and continued till the 4th January. In the space of 47 days seven ounces and a half of belladonna, five grains and a half of oxymuriate of mercury, and four ounces of mercurial liniment had been used. The mercury excited febrile irritation, and the belladonna occasioned vertigo, vomiting, tremors of the limbs, obscurity of vision, &c. The sight of the bath occasioned great anxiety, and sense of constriction about the fauces.

In the second patient there were two wounds in the neck, and several others in the arm. The former were healed on the 4th of November, the latter on the 9th. He felt an obtuse pain and want of power in the arm. On the 19th the warm bath and mercurial liniment were ordered, with belladonna and calomel, internally. By the 30th of the same month, the dose of the belladonna had been increased from about 16 grains to 36 in the day. The calomel and liniment had produced salivation by the 26th of November. The mercurials and the belladonna were continued more or less till the 4th of January. The warm bath could not long be used, the sight of the water producing great oppression about the præcordia, and sense of suffocation at the moment of immersion. The patient experienced also a degree of repugnance to the ingurgitation of watery fluid for several days.

In the case of the third patient, the wounds inflicted by the rabid animal healed on the 7th November. On the 19th, the belladonna, mercury, and warm bathing, were commenced, and continued till the 31st December, when all medicines were discontinued. There was no dread of water in this case.

The fourth of the fortunate patients was bitten in the right arm, in the hand, and in the left hip. The wounds were healed by the 18th of November. His wife and child fell victims to the dreadful disease. He began with two grains of belladonna every four hours, and a drachm of mercurial liniment for daily friction. The belladonna was increased gradually to three drachms in the 24 hours. Slight salivation came on about the 26th November, and the daily use of the warm bath was now added to the other remedies. The whole of the means were continued till the 5th January. In the course of the treatment the patient experienced, besides the usual effects of the belladonna, violent palpitations at night, with cold chills, succeeded by icteritious symptoms, especially in the eyes. On the 21st December he had some hydrophobic symptoms, and complained of pain in the cicatrices of the wounds in the thigh. He peremptorily refused to go into the warm bath; but being forced in, he was attacked by such a degree of anxiety, and also convulsions, that it was necessary to withdraw him immediately.

The subjects of the foregoing narrative continued in health down to the time of writing the memoir, which must have been six or seven years. The other cases all proved fatal, and need not occupy space here. As a prophylactic, we believe mercury to ptyalism is more efficacious than any other remedy exhibited internally; but as we consider the removal of the bitten part as the only thing to be depended on, so when that is done nothing else is necessary. There are circumstances, of course, where the parts cannot be removed, and there are too many instances where excision is neglected;—here the prophylactic is rationally indicated.

The readers of our first series, vol. i. p. 494, *et seq.* will remember a very interesting paper on this subject, by Mr. Daniel Johnson of Torrington, in Devonshire. That gentleman had served a great many years in India, where canine madness is unfortunately very prevalent, and he asserts that—"in every instance where he had time or permission to impregnate the system with mercury, after the infliction of the bite, and before the symptoms of hydrophobia shewed themselves, the latter were entirely prevented."* For many interesting details respecting the mercurial prophylactic, we must refer to Mr. Johnson's paper.

---

30. *Obliteration of the External Iliac.*† Mr. Bryant was called to a woman on the 21st of January of the present year, who complained of acute pain in the *right* hypogastric and iliac regions, increased on pressure, and accompanied by quick, full pulse, costive bowels, and pyrexia in general. These symptoms were judiciously treated by venesection and other antiphlogistic measures, and by the 26th the pain in the iliac region had ceased; but she had great pain in the course of the *left* ureter, with much suffering in making water. These symptoms were also mitigated by appropriate means; but on the 28th, while in the warm bath, she complained of a sudden numbness and coldness of the *left* extremity, succeeded by severe pain in the calf of the leg. These symptoms continued till the 1st February, when petechial spots appeared on the foot, with coldness, and complete loss of sensation. The calf of the leg, however, was swollen and tender. No pulse to be felt in the femoral artery. On the 3d Mr. Brodie saw the patient, and ordered a flan-

* No. 4, for April 1819.

† Mr. Bryant. Med. Repos. Aug. 1822.

nel roller from the foot to the groin. She has diarrhœa. On the 5th Messrs. Brodie, Cline, and Clarke saw the patient. On the 6th, the pulse was 140 and irregular—the foot extensively livid—"the line of separation between the muscular and tendinous portions of the gastrocnemii muscles is faintly discernible." The *right* leg is also very painful. On the 8th the livid appearance had spread farther up the left leg, and vesicles were observable two inches above the ankle. We cannot pursue the diurnal reports of this afflicting case; but we find that on the 20th "the toes of the left foot are shrunk, dry, and withered." On the 9th March, "the foot is fast separating. She is looked upon as hopeless." On the 16th "sphacelation has increased rapidly;" but the pain is more moderate; the bowels regular—pulse 110—appetite improved—sleeps better. 21st. The bones are exposed—rests well—strength improves. On the 6th July, we find that the foot had been removed, merely by dividing the bones, and the fibula was still exfoliating one-third below the knee. The patient's health was out of all danger.

We fully agree with the gentlemen who had the charge of this case, that it was far better to *follow* the steps of Nature, and remove the bones as they became denuded, than to attempt amputation above, when the soft parts were swollen and painful, and there was every reason to believe that the artery was obliterated, and consequently the parts precariously supplied with blood.

---

*Cruritis, or Phlegmasia Dolens.* Dr. Hosack, of New York, has published some cases and observations on this painful disease, in a letter to Dr. Francis of the same city. The cases we shall pass over, as containing nothing very remarkable; but we shall give the Professor's deductions respecting the pathology and treatment of the disease, in his own words. He infers—

"1. That cruritis is an inflammatory disease, not only affecting the limb, but the whole system.

"2. That it most usually proceeds from a suppression of the natural excretions, the effect of cold, stimulating drinks, and other means of excitement.

"3. That it is not necessarily connected with the lochial discharge, as inculcated by Trye, Denman, and, indeed, by Rodrigus Decastro, of Hamburgh, in 1603, by Wiseman, in 1676, and by Mauriceau, in 1712, who were the authors of this doctrine.

"4. That the first irritations frequently appear about the calf of the leg, and not in the groins and pelvis, as asserted by Dr. Denman.

"5. That it follows easy as well as difficult labours; and, therefore, cannot proceed from the pressure of the child's head upon the edge of the pelvis, rupturing the lymphatics, as supposed by Mr. White.

"6. That it is not a disease confined to the lymphatics, but, as in the cases recorded by Dr. Hull, it appears in every part of the affected limb.

"7. That it is not confined to females, but, as in the cases recorded by Dr. Hull, Dr. Ferriar, Dr. Thomas, Dr. Denmark, and others, it occasionally appears in males.

"8. That, as in gout and rheumatism, when depletion is not actively employed, the inflammation, after appearing in one limb, is in some cases transferred to another.

"9. That it sometimes appears in both limbs at the same time.

"10. That the general means of subduing inflammatory action are the most effectual in removing the *active* stage of this complaint.

"11. That in the second stage of cruritis, in addition to the use of general stimuli and tonics, stimulating spirituous liniments, friction, and the roller, are most useful in restoring the circulation, and in exciting the absorbents in the removal of the swelling which remains in the passive stage of this disease.

"12. That occasionally, as in the cases related by Hull, Denman, and by Zinn, it ends in abscess, and proves fatal, especially where the antiphlogistic treatment has not been vigorously pursued in the first stage of the disease, or when it occurs under great exhaustion and debility of constitution."

---

32. *Poisoning by Opium.** In the last number of the Repository, Mr. Sprague, an intelligent practitioner of Kingston on Thames, has laid down some plain, practical, and, we think, judicious rules for the guidance of the medical attendant in cases which do not afford much time for reflection. The first object, of course, is to evacuate the poison. For this purpose Mr. Sprague affirms that the following form of emetic is preferable to all others. ℞. Subcarb. ammoniæ ℈j. pulv. rad. ipecac. ʒss. aquæ menthæ pip. ℥iij. tinct. capsici ʒij. ℳ. ft. haust. emet. This, if deglutition be impracticable, must be conveyed by a tube into the stomach. A little of the liquor ammoniæ on a feather is to be applied to the nostrils, and a more dilute stimulant of the same kind, is to be applied to the external canthus of the eye. The patient's head should be kept raised, and wetted with cold water; but our author does not mention the cold affusion, as recommended by Mr. Wray and Dr. Copland. When the poison is well evacuated, then the vegetable acids are to be employed; and subsequently the exhaustion is to be counteracted by suitable means.

---

33. *Double Hare-Lip, with separated Palate Bones.*† The patient was about twelve years of age—palate bones much separated, and free communications between the mouth and nostrils—con-

---

* Mr. Sprague. Med. Repos.

† Mr. Harrison. Med. Repos. 101.

siderable fissures in the upper lip and jaw bone. Between the fissures was a portion of bone containing two incisor teeth, and connected to the end of the nose by a strong cartilage. This projecting portion of bone appeared to keep the palate bones much apart. It was but slightly covered by integuments, which were indurated by repeated attempts made to unite the side portions of lip to this scanty strip of skin. Mr. Harrison determined to remove entirely both it and the piece of bone containing the two teeth; and effected his object with the metacarpal saw, having first separated, with a scalpel, the integument and cartilage from the nose. This finished, he observed the palate to be more complete, the canine teeth to be more in the front of the mouth, and the circle of the upper jaw lessened. The end of the nose, which had been bound down, obtained freedom and a more natural shape. He found that there was very little want of substance in the lip, after paring the edges, which were readily brought together, and kept in union by pins and the twisted suture. He applied adhesive straps over small cushions, to bring forward the cheeks. The little patient took nourishment through a tube introduced at the angle of the mouth. In five days the pins were removed, and in a very short time the boy was perfectly well.

---

34. *Pathology of the Brain in Mania, &c.** The exceptions to general rules are undoubtedly more numerous in medicine than in any other science. Idiosyncrasy of constitution so modifies the phenomena of the same disease, as to leave it scarcely cognizable, or assignable to the same class, in different individuals. This is the grand cause of medicine being termed reproachfully a "conjectural art." The reproach does not *rightfully* lie at the door of the physician, but of the disease. If the same causes uniformly produced the same effects—or if there was a possibility of drawing general conclusions as legitimately in medicine, as in other sciences, then indeed we might be *justly* reproached as a faculty of conjecturers. But this is not the case. We have a PROTEUS to deal with, in every disease, which assumes as many forms as there are individuals in the world! Those therefore who sneer at the "conjectural art," are generally those who do not know, or do not consider the difficulties attending it:—and, at all events, even *these* will fly to it for aid, whenever the evil hour comes, as come it must to all. Mean time the accumulation of facts, and the more diligent study of morbid anatomy, are daily clearing away the rubbish of conjecture with which the healing art has been defaced since its first institution.

Dr. Hebreart has had excellent opportunities of examining the organ of thought at the Bicetre, where so many maniacs are col-

---

* Observations sur quelques Maladies du Cervelet, du Cerveau, et de leurs Membranes, Recuellies à l'Hospice de BICETRE. Par M. Hebreart Medecin Ord. des Alienées. ANNUAIRE MED. CHIRURGICALE.

lected. He has been able to correct some errors into which physicians have fallen, especially respecting the cerebellum, the diseases of which have generally been considered as very speedily fatal. Our author has accumulated several facts at the Bicetre, which prove that organic affections of the cerebellum may pass into the chronic state, the same as those of other organs, and not destroy life till after a long period of suffering. Again, it is pretty generally supposed that inflammation of the meninges of the brain can hardly exist without producing the phenomena of phrenitis. Yet out author has proved by dissections, 1st, that in persons who exhibited all the symptoms of phrenitis, no morbid appearances could be detected in the meninges;—and 2dly, that in cases where the said membranes were found greatly disorganized, no phenomena of phrenitis were apparent during life. These cases are brought forward not to discourage enquiry, but to check presumption, and thus to save the physician from the disgrace of a false prognosis into which he is not seldom led by too great propensity to generalize, without considering the numerous exceptions to all general rules in medicine.

### AFFECTIONS OF THE CEREBELLUM.

*Case* 1. A young man fell into a state of idiotism, six years before the date of report, after a course of severe study. He then, during the winter, became so debilitated and scorbutic, that he sunk, exhibiting, for the last month of his life, that prostration of strength and aridity of the tongue and lips which characterize the low fevers. On dissection, the four ventricles of the brain were found distended with serous effusion. The parietes of the fourth ventricle were disorganized, having degenerated into a yellow and lardaceous substance, that extended more than a line, in all directions, into the cerebellum.

*Case* 2. A maniac in the Bicêtre, who had, for several years, been considered as incurable, was seized with a complaint which our author looked upon as a common gastric affection, but soon put on the character of low fever, as great prostration of strength, dry and red tongue, encrusted teeth, &c. This state lasted four months, in despite of tonic and stimulant treatment, which produced no amelioration of the symptoms. The patient at length sunk, having the rattles in his throat for eight days before his death. On dissection, a disorganized portion of cerebellum, six lines in diameter, situated at the inferior part of the right lobe, was observed, of a hard consistence, lardaceous, and yellow colour, occupying not merely the cortical, but extending several lines into the medullary substance of the cerebellum. Opposite to this diseased portion the pia mater was destroyed, and the dura mater partook of the yellow appearance of the disorganized cerebellum.

The morbid structure in this and the preceding case, our author rationally imagines to have been of considerable standing. They were probably, he thinks, in both cases, the cause of the insanity.

*Case* 3. A man of strong constitution, a sawyer of marble, (an occupation not very much calculated to excite the passions,) experienced an accession of mania in 1811, he being then in his 50th year. This attack lasted three months without any lucid interval. Partly by time, partly by the lowering plan of treatment pursued at the Bicetre, the malady was overcome, and the patient returned to his wife and his former trade, having been six months in the hospital. He continued sane for two years, when he was again brought to the Bicetre labouring under a milder form of mental derangement than before; but considerably debilitated in physical power, without any apparent disorder of the great functions of the body. His speech was faltering—his tongue slightly paralyzed—his memory obliterated. This state continued several months, and then gradually changed into a kind of low fever characterized by dryness of the tongue, incrustations of the teeth, and general prostration of strength, which symptoms resisted all the tonics and stimulants that could be employed. The debility gradually increased, with scarcely any deviation from a natural state in the pulse or animal temperature—in short, without any of that reaction which is generally observable even in the lowest fevers. The patient at length ceased to exist, after lying fifty-five days without speaking a word, without turning in his bed, without exhibiting any nervous movement or symptom of irritation.

On dissection, the thoracic and abdominal viscera presented nothing remarkable. In the head, all the parts were infiltrated with black blood. (" Toutes les parties infiltrés d'un sang noiratre.") The lateral ventricles were distended by a considerable quantity of water; but what was most remarkable was a gelatinous degeneration in the left lobe of the cerebellum. All the inferior surface of this lobe had the consistence of jelly to the depth of three or four lines. It was only necessary to scrape the parts gently with the handle of the scalpel, in order to reduce them to a fluid. When this was done, the lobe, instead of presenting a convex surface, had a concave aspect, the parietes of which concavity were hard, polished, and formed a kind of cyst. Our author queries if it be not probable that the attack of insanity, in 1811, was owing to inflammation in this part of the cerebellum, which becoming chronic, produced at length the alteration of texture above described.

### CEREBRUM.

Affections of the *brain* are much more common in maniacal cases than of the cerebellum.

*Case* 4. A soldier, 32 years of age, of sanguineous temperament, was admitted as insane into the Bicetre. He had been rendered deaf by exposure to a long and tremendous cannonade, after which he showed symptoms of mental alienation. He was attacked by phthisis pulmonalis while in the Bicetre, and after going through the different stages, died. On dissection, the viscera of the abdomen were found healthy—the lungs filled with tubercles in a state of suppuration—the meninges of the brain healthy—a tumour the size of a nut in the anterior lobe of the right hemisphere. This tumour was enveloped in a peculiar membrane or cyst, intimately connected with the surrounding cerebral mass. Cut into, the tumour presented a substance of a lardaceous consistence.

The two next cases related by our author were epileptics, between whom and maniacs there is but too much relationship.

*Case* 5. A man, aged 40, had been epileptic since the age of 18, the complaint having been brought on by a fright. The paroxysms of the disease were not, in general, more frequent than once or twice in the month. During one night, however, while in the Bicetre, he had several paroxysms of epilepsy in succession, and no alarm being given by the nurses, the man died with all the symptoms of asphyxia—an accident which not uncommonly happens, under such circumstance, unless bleeding be had recourse to in time. On dissection, a portion of the posterior lobe of the left hemisphere of the brain was found disorganized, and converted into a yellow pultaceous matter, and surrounded by a portion of indurated brain.

Another epileptic, 30 years of age, had had paroxysms of the disease twice a week for the last five years; the disease having arisen without ostensible cause, and not as yet attended by intellectual aberration. He was suddenly seized one day, without evincing (according to the report of the nurse) any of those struggles which attended all former paroxysms, and carried to the infirmary of the institution in the following state:—face flushed—vessels of the conjunctiva gorged—tongue embarrassed—ideas confused—pulse strong and frequent—respiration sibilant. Notwithstanding bleedings, sinapisms, and other means, the apoplectic state increased, and the patient died on the third day of the attack.

On dissection, the viscera of the thorax and abdomen were found healthy, with the exception of the heart, which was very large in proportion to the size of the patient. The cerebral vessels were greatly gorged with blood. On the borders of the hemispheres opposite the falx, were developed, very gradually no doubt, red, callous excrescences, similar in appearance to those syphilitic vegetations called *cauliflower* excrescences. The lateral ventricles, especially the left, were very much distended with serous effusion.

The following case is remarkable.

*Case* 7. Francis ———, 56 years of age, who had been three years in prison, evinced, towards the end of November 1814, a certain absence of mind, which was remarked by his comrades. On the 1st December, he experienced a sudden attack of general paralysis, and was carried to the infirmary, being incapable of speaking, hearing, seeing, or feeling—the muscles were relaxed, the respiration natural, the animal temperature moderate, the pulse slow and not very strong. Four grains of emetic tartar were exhibited, but produced no effect. In the evening four grains more were given, which produced only a few stools. He now swallowed, though with difficulty, some stimulating potions—sinapisms were applied to the feet. It was not till after 48 hours that he recovered from this lethargic state resembling a profound sleep. Seven hours after this, he experienced an attack of mania, which lasted but a few hours, and then changed into the original lethargy, in which he lay insensible. After applying blisters to the thighs, and taking a large quantity of blood from the arm, the patient recovered sense again for nine or ten hours, after which he relapsed once more into a state of *carus*, as above described. During the lucid interval he complained greatly of his head, and of pains in his limbs. He continued to relapse and revive alternately, till the 15th December, when he found himself so well as to be able to take some food. He had no other attack till the 21st, when he fell out of a window into the street, and was taken up paralytic, as in the first instance. This state continued two days, during which he was insensible. From this he revived, and was rather violently insane for some hours, when he fell back into *carus*, which lasted 36 hours. This state of alternate carus and mania continued till the 12th January, 1815. The perodicity of the attacks, and the want of well marked pyrexia, determined our author to exhibit the cinchona, both in powder and decoction. In three days from the commencement of this mode of treatmeut, the symptoms, both of mania and paralysis, disappeared. The medicine was continued five days, and the man was so well in a month, as to return to his domestic avocations.

Our author has no doubt in his own mind, that the above was a case of masked intermittent fever of a malignant nature—"*fievre intermittente pernicieuse larvée.*"

### MENINGES.

There can be no doubt that, in the great majority of cases, inflammation of the cerebral meninges will exhibit the usual phenomena of phrenitis—as fever, exaltation of the intellectual functions, delirium, and functional disturbance in various other organs than the brain. The following observations are brought forward to prove that the rule is not absolute, and that the phrenitic excitement is not always in proportion to the meningeal inflammation.

*Case* 8. A professor of music, 47 years of age, from some domestic inquietude, fell gradually into the following state:—agitation and delirium—insomnium—physical sensibility greatly increased—hard and full pulse—skin moist. If not confined, the patient was constantly in motion, upsetting and breaking every thing in his way. When confined by means of the straight waistcoat, &c. he struggled incessantly to disengage his limbs. He only pronounced some incoherent words and sentences, showing that his ideas were as disorderly as his muscular movements. After continuing nine days in this state, an inflammation arose in his right leg, which quickly took a gangrenous turn from the continual friction of the part, in consequence of the patient's restlessness. He died on the 17th day from the invasion of the phrenitic state.

Dissection was confidently expected to disclose well marked phlogosis of the brain or its membranes. No such thing was found. The contents of the cranium, and of both the other great cavities, were perfectly natural, with the exception of a rather firmer consistence than usual of the muscular substance of the heart, and of the brain and spinal marrow.

That the death of this patient was accelerated by the gangrene of the leg, there can hardly be a doubt; yet it is abundantly evident from this case, and still more so from the one which follows, that such disturbance of *function* may take place in the brain, as may destroy reason and even life, without producing any cognizable alteration of structure.

*Case* 9. A farmer, 53 years of age, lean, and accustomed to hard work, experienced, during the troubles of 1813, the loss of all his little property, and was himself obliged to fly to the woods to preserve his life. On returning to his former habitation, and finding but a wilderness, he lost his appetite, became sleepless and inconsolable, notwithstanding the incessant exertions of his children to cheer him up. His intellects soon gave way, and his family were forced to convey him to the Bicêtre. Here he was exceedingly violent, biting, tearing, and fighting with his keepers—crying out incessantly, and refusing all sustenance, or swallowing it at times voraciously. He continued fifteen days in this state, his strength gradually declining, but the insanity remaining unmitigated. The tongue to the last was moist, the pulse natural. On dissection, no inflammation, or traces of such, could be detected in any part of the brain or its coverings—on the contrary, they appeared blanched, and, as it were, deprived of their due share of the vital fluid. No disease in the thoracic or abdominal viscera.

In respect to the above case, it is but fair to bear in mind that the abstinence from food, and gradual extenuation of the body before death, might very considerably tend to prevent post mortem traces of inflammation being cognizable, even if they had actually existed during life. We are of opinion indeed that *simple inflammation* of a serous tissue is often obliterated by death, or rather the exhaustion which precedes death. It is one or other of the terminations of inflammatory action, which remain indelible, such as effusion, thickening, purulent secretion, &c. When the usual symptoms of inflammation are very evident during life, and yet no change of structure can be perceived on dissection, we are hardly justifiable in denying the pre-existence of phlogosis—and still less would we be justifiable in acting on this supposition in practice. The external signs of inflammation must be combated by the usual means, regardless of the *possibility* of our having a neurosis instead of a phlogosis to deal with.* Still it is very proper and necessary to

* As far as our own observations have extended, we have been led to think moral causes act more on the functions, and physical causes on be acquainted with the exceptions as well as the general rules—especially in medicine, where they are so numerous.

The following case is fully as remarkable as its predecessors.

*Case* 10. A man was picked up by the police, and sent to the Bicêtre as alienated. No history could be traced, nor even could his name be ascertained. He appeared to be about 30 years of age, and muscular. He spent four days in the hospital in the following state:—he was constantly standing upright, his head thrown backwards—his limbs were on the stretch, but not stiff—he frequently uttered the following exclamation, "*Ah! mon Dieu!*" followed by a muttering of some indistinct words—his eyes were prominent and always open—the conjunctivæ injected—the pupils but little dilated, and not affected by light or shade—the jaws were kept shut, but not spasmodically locked—drink introduced into the mouth passed out again, without being swallowed. The physical sensibility was as obtuse as the moral, for he felt neither the pricking of pins, nor the aspersion of cold water, nor the application of heat, although blisters rose when applied to the skin. The organs of sense were not entirely obliterated in function, but external impressions on them could not rouse the brain to any reaction. Attention and judgment were completely suspended. The pulse was full and hard. A venesection made no alteration in the symptoms for better or worse. He died suddenly on the fourth night from his entrance into the Bicêtre; and on dissection the brain and its membranes, as also the thoracic and abdominal viscera, were found in a state of perfect integrity.

We have but one more case to analyse, and then we shall close this article.

*Case* 11. A man, 37 years of age, thin, and of a bilious temperament, was arrested for political opinions, and kept three months in prison, when he was acquitted. The doors of his jail had scarcely been opened, when he evinced a high degree of mental excitement—ran home and broke every thing that came in his way—knew not his parents—and would have thrown himself out of the windows, had they not been well guarded. He was conducted to the Bicêtre. His pulse was nervous, feeble, and frequent—he was constantly crying out, and muttering words without sense—his eyes were fixed—a cold perspiration covered the body—his agitation was without remission, day and night, in spite of sedative, and antispasmodic potions, warm baths, sanguineous evacuations, sinapisms, and blisters. He died on the third day after coming to the hospital, and on the fifth from the commencement of the phrensy. On dissection, the muscles of the extremities appeared in a state of rigid contrac-

---

the structure of the brain. It is therefore very advisable, in all cases, to enquire particularly into the etiology of cerebral affections, and into the manners, habits, and tempers of the patients.

tion—the heart was rather larger than natural, and firm—stomach and intestines contracted—all parts of the brain firm, and of the consistence of liver, so as to crepitate under the scalpel. Outwardly and inwardly the brain was of a white colour. No fluid was found in the ventricles, and the meninges were perfectly healthy.

This firmness of the brain has been observed in many maniacs, and was pointed out by Morgagni in epileptics. We hope the above facts will be found useful, and we shall, for the present, make no comments on them.

